# Zwitterionic Mn(III)‐Tetraphenyl Porphyrins: Water‐Soluble MRI Contrast Agents with High Relaxivity

**DOI:** 10.1002/cmdc.70268

**Published:** 2026-04-16

**Authors:** Darius Ludolfs, Lennart F. V. Spickschen, Stefanie Bredehöft, Verena R. Schulze, Marie Oest, Samila Leon Chaviano, Neus Feliu, Markus Fischer, Marc‐André Fortin, John V. Frangioni, Wolfgang Maison

**Affiliations:** ^1^ Department of Chemistry Institute of Pharmacy Universität Hamburg Hamburg Germany; ^2^ Fraunhofer Institute of Applied Polymer Research IAP Center of Applied Nanotechnology CAN Hamburg Germany; ^3^ Hamburg School of Food Science Institute of Food Chemistry Universität Hamburg Hamburg Germany; ^4^ Axe Oncologie Centre de Recherche du CHU de Québec – Université Laval Québec Canada; ^5^ Centre de Recherche sur le Cancer (CRC) de l’Université Laval Québec Canada; ^6^ Département de Génie des Mines de la Métallurgie et des Matériaux Université Laval Québec Canada; ^7^ Curadel Pharma Bonita Springs FL USA

**Keywords:** contrast agents, magnetic resonance imaging, manganese‐based contrast agents, porphyrins, zwitterions

## Abstract

Manganese‐based contrast agents (MBCAs) are promising alternatives to currently used gadolinium‐based contrast agents (GBCAs) for magnetic resonance imaging (MRI). This study describes the synthesis and physicochemical evaluation of two new zwitterionic Mn(III) porphyrin chelates (**Mn‐8** and **Mn‐9**). Both compounds were synthesized *via* copper‐catalyzed azide–alkyne cycloaddition (CuAAC) from an azido‐substituted precursor. Both zwitterionic compounds are soluble in water and are remarkably stable to acidic pH and transmetallation under challenging conditions. The complexes have a high *T*_1_‐relaxivity, comparable to current clinical high‐relaxivity GBCAs like gadopiclenol. In addition, zwitterionic complexes **Mn‐8** and **Mn‐9** have superior relaxivity and improved stability compared to Mn‐DPDP (mangafodipir, Teslascan). The favorable properties of both compounds can be attributed to the decoration of the chelator with sulfobetaines or *N*‐oxides. Importantly, chelation of Mn(III) by the porphyrin drastically reduces singlet oxygen generation. **Mn‐8** showed good contrast enhancement in vivo. These compounds are thus strong candidates for the development of next‐generation gadolinium‐free MRI contrast agents.

## Graphical abstract

Mn(III)‐based contrast agents (MBCAs) are promising and potentially safer alternatives to the Gd(III)‐based contrast agents (GBCAs) currently used in MRI. This study reports the synthesis, physicochemical characterization and first in vivo application of novel Mn(III)‐tetraphenylporphyrin chelates, bearing zwitterionic sulfobetaine or *N*‐oxide groups.

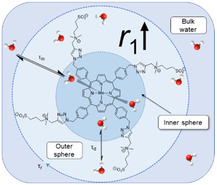

## 1 Introduction

With about 40 million doses per year, contrast agents play a crucial role in *T*_1_‐weighted contrast‐enhanced magnetic resonance imaging (CE‐MRI) [1, 2]. They lead to enhanced contrast between healthy and diseased tissue by accelerating proton relaxation. Currently, gadolinium‐based contrast agents (GBCAs) are used clinically for this purpose. With its half‐filled *f* ^7^ shell (seven unpaired electrons), Gd(III) shortens the proton *T*_1_ relaxation time of protons in neighboring water molecules [3]. However, Gd(III) is a toxic lanthanide ion and can therefore be used in vivo in the form of stable Gd(III)‐complexes only [4, 5]. The release of small amounts of Gd(III) has led to restrictions for the use of some GBCAs by regulatory authorities [6, 7, 8]. The accumulation of intact and stable Gd(III) complexes [9, 10] particularly in the kidney [11] has also raised concern regarding the safety of GBCAs in recent years [12, 13].

The development of gadolinium‐free paramagnetic contrast agents has thus gained interest [14]. In this context, manganese is an interesting candidate for gadolinium substitution [15, 16, 17, 18, 19]. Depending on its oxidation state, it has four (Mn(III)) or five (Mn(II)) unpaired electrons and can therefore efficiently enhance proton relaxation. As an essential element, manganese ions are considered to be less toxic than Gd(III). However, larger quantities of manganese ions are neurotoxic in humans [20]. Just like Gd(III), Mn(II) and Mn(III) should thus be formulated as stable complexes for in vivo application.

**FIGURE 1:**
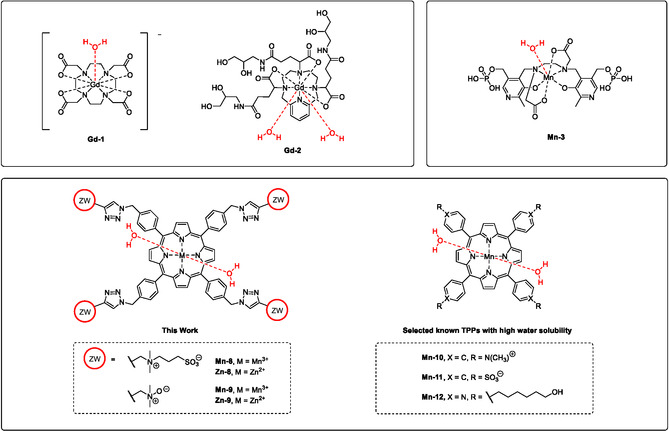
Structure of water soluble Mn(III)‐TPPs **Mn‐8** and **Mn‐9**, their corresponding Zn(II) analogues **Zn‐8** and **Zn‐9**, as well as GBCAs Gd‐DOTA **Gd‐1** [21], gadopiclenol **Gd‐2** [22] and MBCA Mn‐DPDP **Mn‐3** [23]. Known examples of water soluble Mn(III)‐TPPs include compounds **Mn‐10** [24], **Mn‐11** [25] and **Mn‐12** [26].

The first clinically used manganese‐based contrast agent (MBCA) was Mn‐DPDP **Mn‐3** (Figure 1). Mn‐DPDP is a Mn(II) complex of the acyclic chelator dipyridoxin‐diphosphate (DPDP) and was used as a liver‐specific contrast agent [27, 28, 29, 30]. The complex has limited stability in vivo and was withdrawn from the market due to concerns regarding the release of potentially neurotoxic Mn(II) in patients. Complex stability is therefore a critical point for the development of new MBCAs [31, 32, 33]. Among the most promising MBCAs developed is Mn(II)‐PyC3A, which has been demonstrated to combine good stability with a favorable biodistribution for many MRI applications [19, 34]. Porphyrin complexes of Mn(III) have also attracted interest in this context. Mn(III) fits well into the coordinating plane of the macrocyclic chelator, resulting in stable complexes [35]. Typically, two water molecules complete the octahedral coordination sphere of Mn(III) in these complexes [35]. As a consequence of the inner‐spere hydration number (*q* = 2), some Mn(III)‐porphyrins have longitudinal relaxivities significantly higher than most common GBCAs (with typically *q* = 1, e.g. gadoteric acid **Gd‐1**) and MBCAs such as Mn(II)‐DPDP and Mn(II)‐PyC3A [34, 36]. A notable exception among GBCAs is gadopiclenol **Gd‐2**, which has a higher relaxivity than many other GBCAs due to a hydration number of *q* = 2 and hydrophilic isoserinol sidechains [37].

A common drawback of Mn(III)‐porphyrins is their poor solubility in water. Efforts to increase the solubility of porphyrins include the addition of cationic (e.g. ammonium) or anionic (e.g. carboxylate) charged groups [26, 36]. Information about toxicity or biodistribution of these compounds is limited, but problematic properties of charged small molecules are well known: cationic molecules can cause cytotoxicity by destabilization of cell membranes [38, 39, 40]. Anionic groups are generally considered to be more biocompatible, but anionic drugs tend to be accumulated in liver and spleen. This is mainly due to uptake by Kupffer cells as well as organic anion transporters [41, 42]. Other approaches to increase water solubility without adversely affecting pharmacokinetics are therefore needed. The ‘decoration’ of contrast agents with zwitterionic groups such as sulfobetaines or *N*‐oxides is particularly interesting in this context and has led to the development of GBCAs with high relaxivity [43, 44, 45]. These reagents are particularly valuable due to favorable pharmacokinetic properties because zwitterions can prevent unwanted tissue retention of the drugs [45, 46, 47, 48].

The aim of this study was the development of Mn‐(III)‐porphyrin complexes with zwitterionic groups (sulfobetaines or *N*‐oxides). A modular synthesis of different zwitterionic tetraphenylporphyrin (TPP) Mn(III)‐complexes **Mn‐8** and **Mn‐9** via copper‐catalyzed azide–alkyne cycloaddition (CuAAC) was developed, the resulting complexes were analyzed with respect to their physicochemical properties, longitudinal relaxation rates (1/*T*_1_) at 1.4 T, and the stability of the complexes under acidic conditions and transchelation challenge. Furthermore, the generation of reactive singlet oxygen was evaluated, and the MRI contrast enhancement of **Mn‐8** was measured in mice.

## 2 Results and Discussion

### 2.1 Synthesis of Mn(III) Complexes

**SCHEME 1:**
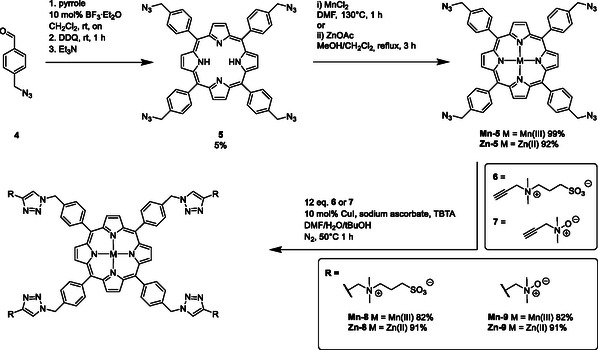
Synthesis of zwitterionic TPP‐complexes.

The synthesis of zwitterionic MBCAs is depicted in Scheme 1. Azidomethylbenzaldehyde **4** was prepared via nucleophilic substitution of 4‐bromomethylbenzaldehyde with sodium azide in DMF. Clickable porphyrins were synthesized following the procedure of Le Pleux et al*.* [49]. Condensation of aldehyde **4** with freshly distilled pyrrole in dry CH_2_Cl_2_ was catalyzed by 10 mol% BF_3_·Et_2_O. Subsequent oxidation with DDQ (2,3‐dichlor‐5,6‐dicyano‐1,4‐benzoquinone) gave tetraazido‐TPP **5**. This type of conversion is known to give generally low yields [50]. However, it is the easiest approach to substituted TPPs. Efforts to convert **5** via CuAAC were not successful due to the immediate formation of Cu(II)‐porphyrin complexes. The tetraazide **5** was thus first converted to the corresponding manganese complex **Mn‐5.** In addition, the Zn complex **Zn‐5** was prepared as a reference compound for transchelation assays. Subsequent CuAAC of the complexes **Mn‐5** and **Zn‐5** were performed with 12 equivalents of alkyne **6** [51] or **7** [52] in a mixture of DMF/H_2_O/*t*BuOH (2:1:1). Copper(I) iodide, sodium ascorbate, and (TBTA) Tris[(1‐benzyl‐1H‐1,2,3‐triazol‐4‐yl)methyl]amine were used in concentrations of 10 mol% respectively, following an established protocol [53]. No transchelation of the Mn‐ and Zn‐complexes to the corresponding copper complexes was observed in these conversions by analysis via LC/MS. Residual copper was removed by stirring of an aqueous solution of the target compound over QuadraPure TU resin for 48 h. Remaining copper concentrations were lower than 0.01% as verified by inductively coupled plasma mass spectrometry (ICP‐MS) analysis. LC/MS analysis of the CuAAC to the *N*‐oxides **Mn‐9** and **Zn‐9** revealed the formation of side products due to a copper‐mediated partial reduction of *N*‐oxide groups [54]. This unwanted side reaction was avoided by short reaction times of 1 h. All zwitterionic complexes obtained by this procedure had good water solubility up to 0.5 m.

### 2.2 Relaxivity Measurements

**FIGURE 2:**
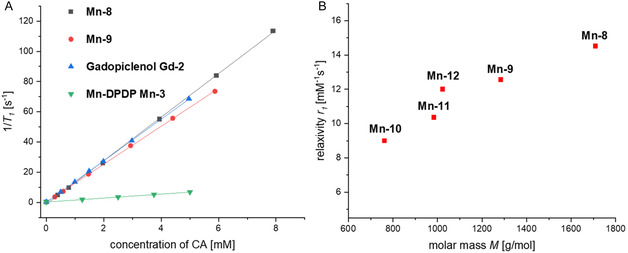
(A) Plot of the longitudinal relaxation rate 1/*T*_1_ against the concentration of contrast agents: Mn(III)‐TPPs **Mn‐8** and **Mn‐9**, gadopiclenol **Gd‐2** and Mn‐DPDP **Mn‐3**. Single‐point measurements have been performed at 60 MHz (1.4 T) in H_2_O at 37°C. (B) Dependence of the longitudinal relaxivity (1.4 T, H_2_O, 37°C) on molecular mass of selected structurally related Mn–TPP complexes.

Higher *T*_1_‐relaxivities of GBCAs induce greater relaxation rate changes at equal concentrations, enhancing *T*_1_‐weighted image contrast more effectively [55, 56]. High‐relaxivity contrast agents can thus be used in lower dose and may lead to less retention of toxic metal in vivo. The longitudinal relaxivity of the Mn‐TPPs **Mn‐8** and **Mn‐9** was determined in aqueous solution (1.4 T, 37°C, Figure 2A). The relaxation time *T*_1_ of the zwitterionic complexes, gadopiclenol and Mn‐DPDP was measured using a standard inversion‐recovery pulse sequence. All concentrations were precisely determined by ICP‐MS. The relaxivity value *r*_1_ of **Mn‐8** was measured to be 14.5 mm^−1^s^−1^ (*r*_2_ = 18.4 mm^−1^s^−1^). For **Mn‐9** *r*_1_ was 12.6 mm^−1^s^−1^ (*r*_2_ was 15.5 mm^−1^s^−1^) (Table 1). These values are high compared to most other MBCAs and GBCAs of low molecular weight (**Gd‐1** and **Mn‐3**, for example, have *r*_1_ values of 1–4 mm^−1^s^−1^ at comparable magnetic field strength, see Table 1), but they are comparable to structurally related Mn–TPP complexes of high water solubility (such as **10**, **11,** and **12**) [26]. The latter point is important because more lipophilic Mn(III)‐porphyrins have been found to aggregate in solution, which limits the exchange between bound and solvent water molecules leading to decreased relaxivity values [58, 59]. Both zwitterionic complexes **Mn‐8** and **Mn‐9** were highly water soluble (aqueous stock solutions of both compounds with a concentration of 0.5 m were prepared).

**TABLE 1:** Comparison of molecular mass, *r*_1,_ and hydration number *q* for selected Mn(III) and Gd(III) complexes in water at 37°C.

Compound	Molar mass, g/mol	Field strength, *T*	*r*_1_, mm^−1^s^−1^
**Gd‐1**	580.62	1.5	3.91 ± 0.13^a^ [57]
**Gd‐2**	970.10	1.4	13.82 ± 0.05^b^
**Mn‐3**	689.36	1.4	1.34 ± 0.01^b^
**Mn‐8**	1708.92	1.4	14.52 ± 0.11^b^
**Mn‐9**	1284.36	1.4	12.56 ± 0.08^b^
**Mn‐10**	761.76	1.4	9.00^c^ [26]
**Mn‐11**	983.67	0.47	10.36 ± 0.09 [25]
**Mn‐12**	1023.38	1.4	12.00 [26]

a Measured in human whole blood.

b Standard deviation derived from linear fitting in OriginLab 2025.

c Derived from NMRD data [26].

Among other parameters, the relaxivity of contrast agents depends on the hydration number *q* of the complex, the mean residence time of inner‐sphere water molecules *τ*_m_, the rotational correlation time of the complex *τ*_r,_ and the electronic relaxation time [60, 61]. Overall relaxivity has contributions of inner‐sphere and second‐sphere effects [62, 63]. High *r*_1_ values were observed for the new zwitterionic complexes **Mn‐8** and **Mn‐9** as well as for the structural analogues **Mn‐10**‐**12**. The latter three compounds have been reported previously by Nemeth et al. [26]. The *r*_1_ values of all five compounds **Mn‐8‐12** are in a range expected for water‐soluble Mn(III)‐TPP complexes, but they reveal slight differences. Some factors can most likely be ruled out to explain these differences. The hydration number of various Mn(III)‐TPP complexes is typically *q* = 2, and the water exchange rates are similar among these complexes [26, 64, 65]. Both factors are thus unlikely to contribute to the observed variations in relaxivities. Instead, variations in the hydrodynamic size of the complexes and thus different rotational correlation times can explain the observed values. All Mn complexes **8–12** have the same molecular shape but different molecular weight. Complexes **Mn‐8** and **Mn‐9** have the highest molecular weight and thus the highest relaxivity values (Figure 2B).

It is also notable that the relaxivities of **Mn‐8** and **Mn‐9** are 3.2 and 3.7‐fold higher compared to commercial gadoteric acid (**Gd‐1**) and are comparable to the value of the high‐relaxivity GBCA gadopiclenol (**Gd‐2**) at clinically relevant field strength.

### 2.3 Stability Assays

**FIGURE 3:**
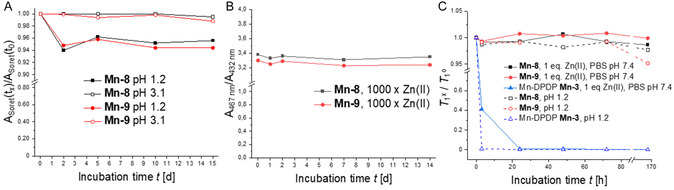
(A) Changes in Soret band intensity at 467 nm of Mn(III)‐TPPs **Mn‐8** and **Mn‐9** following incubation in water at pH 1.2 or pH 3.1 and 37°C. pH was adjusted with HCl. The ratio of measured absorbance A_Soret_(*t*_*x*_) (single measurement) relative to initial absorbance A_Soret_(*t*_0_) was plotted against incubation time *t*. (B) Incubation of **Mn‐8** and **Mn‐9** with Zn(II) chloride at 37°C in PBS (pH = 7.4). The ratio of Mn(III)‐TPP Soret band absorbance relative to Zn(II)‐TPP Soret band absorbance (single measurement) is plotted against incubation time *t*. (C) Evaluation of complex stability *via* changes in longitudinal relaxation time *T*_1_. Mn(III)‐TPPs **Mn‐8**, **Mn‐9,** and Mn‐DPDP **Mn‐3** were incubated in either aqueous HCl at pH 1.2 or in PBS buffer at pH 7.4 containing an equimolar amount of ZnCl_2_ at 37°C. Changes in relaxation time *T*_1_ (single measurement) were plotted against incubation time *t*.

**FIGURE 4:**
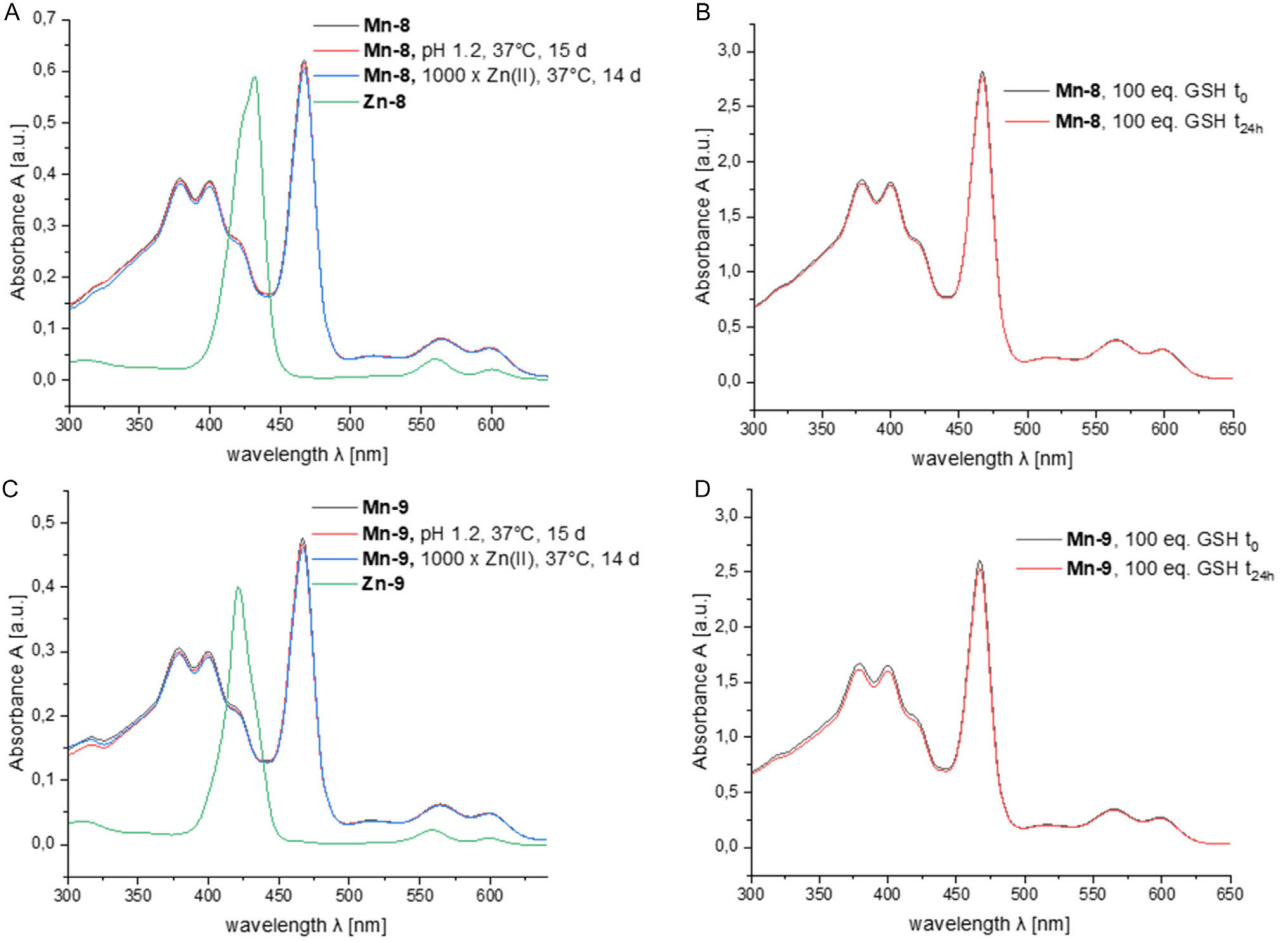
(A) Comparison of UV–vis spectra of Mn(III)‐TPP **Mn‐8** and the corresponding Zn(II)‐TPP **Zn‐8**; (B) Comparison of UV–vis spectra of **Mn‐8** (40 µm) in the presence of GSH (4 mm) following 0  and 24 h incubation in PBS buffer (pH 7.4 at 37°C); (C) Comparison of UV–vis spectra of Mn(III)‐TPP **Mn‐9** and the corresponding Zn(II)‐TPP **Zn‐9**; (D) Comparison of UV–vis spectra of **Mn‐9** (40 µm) in the presence of GSH (4 mm) following 0  and 24 h incubation in PBS buffer (pH 7.4 at 37°C).

Complex stability is a crucial factor for the safe application of MBCAs. A high kinetic stability is a key determinant of the in vivo stability of MBCAs. It determines the resistance of the complex to decomplexation under strongly acidic conditions. The zwitterionic Mn(III)‐TPP complexes were evaluated for stability against decomplexation in acidic solution at pH values of 1.2 and 3.1 at 37°C. The integrity of the complex was monitored by measuring the UV/vis absorbance at 467 nm (Soret band) over time (Figure 3A). It is helpful in this context that the Soret band of Mn‐TPPs is significantly red‐shifted compared to the free chelator TPP and many other metal complexes thereof (see also Figure 4A and C) [26, 66]. Acid‐mediated decomplexation of the complexes leads thus to a decrease in intensity of the Soret band at 467 nm.

Both complexes, **Mn‐8** and **Mn‐9**, revealed a high kinetic stability under acidic conditions for a period of 15 d. The small loss in intensity at 467 nm observed for both compounds at pH 1.2 is most likely not due to decomplexation. Mn(III) porphyrins are known to undergo axial ligand exchange in polar‐protic solvents. In water, the axial positions are typically occupied by two water molecules. Halides readily interchange with these water ligands to form the corresponding halide complexes. This exchange affects the absorption spectrum by a slight redshift of the Soret band and by a decrease in the absorption of **Mn‐8** and **Mn‐9** [67, 68, 69]. The initial decrease in Soret band intensity observed for **6a** and **7a** at low pH is thus most likely due to the exchange of axial water ligands with chloride anions in diluted HCl. We verified this hypothesis by measuring the UV/vis absorbance of **Mn‐8** and **Mn‐9** in aqueous NaCl solution (63 mm) at pH 7. Both compounds showed a comparable 5% decrease in Soret band absorbance in NaCl compared to the measurement in pure water.

The stability of the Mn complexes at pH 1.2 was also monitored by measuring the longitudinal relaxation times *T*_1_ at 1.4 T. Figure 3C shows a plot of the change in longitudinal relaxation time against incubation time. Mn‐DPDP **Mn‐3** has been reported to have limited stability and was therefore analyzed as a control compound. An equimolar solution of Mn(II) chloride at pH 1.2 was selected for endpoint determination. **Mn‐3** showed almost complete dissociation after only 3 h at pH 1.2. In contrast, the zwitterionic Mn‐TPP complexes **Mn‐8** and **Mn‐9** were almost completely stable for up to 168 h at pH 1.2 confirming the results of the spectrophotometric measurements mentioned above.

The thermodynamic stability of the zwitterionic Mn‐TPPs was evaluated using a transmetalation assay with excess Zn(II). Numerous porphyrins are known to form stable complexes with Zn(II) [70, 71]. For colorimetric evaluation, porphyrins **Mn‐8** and **Mn‐9** were incubated with a 1000‐fold excess of Zn(II) in water at 37°C. Figure 3B shows a plot of the ratio of absorbance at 467 nm (Soret band of Mn‐TPPs **Mn‐8** and **Mn‐9**) relative to 432 nm (Soret band of Zn‐TPPs **Zn‐8** and **Zn‐9**) against time. The data confirmed the stability of Mn‐TPPs **Mn‐8** and **Mn‐9** against transmetalation with Zn(II) and revealed no formation of the corresponding Zn(II)‐complexes over 2 weeks. Furthermore, no changes in the UV/vis‐absorbance spectra were visible confirming the integrity of the complexes (Figure 4A and C). These results were confirmed by evaluation of the longitudinal relaxation time over 5 d (Figure 3C). Mn‐DPDP **Mn‐3** was included as a control. The relaxation times remained constant for both zwitterionic complexes **Mn‐8** and **Mn‐9** indicating their stability against transmetalation. In contrast, Mn‐DPDP was unstable under the same conditions and Mn(II) was completely dechelated in less than 24 h. This result confirmed earlier studies on the stability of Mn‐DPDP and was expected given the increased thermodynamic stability of Zn‐DPDP (logK = 19.0) in comparison to Mn‐DPDP (logK = 15.1) [72]. The serum stability of complexes **Mn‐8** and **Mn‐9** was also confirmed. Both complexes were stable over a period of 21 d in human serum at 37°C as measured by LC‐MS (see Figures S28‐S31).

Several Mn(III)‐porphyrin derivatives have been shown to undergo (reversible) reduction to the corresponding Mn(II) complexes under physiological conditions [58, 73, 74, 75, 76]. On one hand, this redox chemistry is a feature that has been exploited for the development of redox‐responsive MRI agents. On the other hand, it could also lead to problematic in vivo toxicity. The conversion of Mn(III)‐TPP to Mn‐(II)‐TPP complexes can be followed by UV–vis spectroscopy [77]. The redox stability of **Mn‐8** and **Mn‐9** was evaluated in the presence of 100 eq glutathione (GSH), and both compounds were found to be stable under these conditions (Figure 4B and C). It should be noted that this finding does not exclude a redox reactivity of **Mn‐8** and **Mn‐9** in vivo because other reductive agents may be relevant here [77]. A more detailed study is needed to thoroughly investigate the in vivo redox properties of the compounds presented herein, which is not the focus of this study.

In summary, the results revealed an excellent thermodynamic and kinetic stability of zwitterionic Mn‐TPPs **Mn‐8** and **Mn‐9**. Unlike Mn‐DPDP **Mn‐3**, the zwitterionic complexes have thus a low potential to release manganese ions in vivo. **Mn‐8** and **Mn‐9** were also stable in human serum and were not reduced by GSH in PBS.

### 2.4 Generation of Singlet Oxygen

Some TPP derivatives have been reported to act as photosensitizers to give reactive singlet oxygen under irradiation with visible light [78, 79]. It is notable in this context that Mn(III)‐TPP's are known to have very low quantum yields leading to drastically reduced singlet oxygen generation compared to other TPP derivatives [80]. However, potential phototoxic effects of zwitterionic TPP derivatives were evaluated with 1,3‐diphenylbenzoisofurane (DPBF) as a probe for singlet oxygen. DPBF undergoes a [4 + 2]‐cycloaddition with singlet oxygen to give an unstable endoperoxide, which readily degrades. This in turn leads to a decrease in absorbance of the DPBF molecule at 415 nm proportionally to the amount of singlet oxygen produced [81, 82].

**FIGURE 5:**
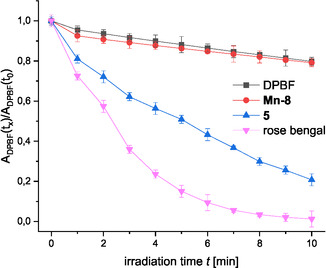
Singlet oxygen‐mediated degradation of DPBF in the presence of photosensitizers rose bengal and TPP **5,** as well as Mn(III)‐TPP **Mn‐8** in DMF. Measurements were done in triplicate. Mean values and standard deviation are depicted.

Porphyrins **5**, **Mn‐8** **,** and the known photosensitizer rose bengal were treated with DPBF and irradiated with a white light LED in a quartz cuvette. The absorbance of DPBF at 415 nm was measured for 10 min and plotted against irradiation time (Figure 5).

While rose bengal and TPP **5** show a clear reduction in DPBF absorbance at 415 nm due to the generation of singlet oxygen, **Mn‐8** does not lead to any singlet‐oxygen formation. These photochemical properties reflect a shorter triplet state lifetime of Mn(III)‐complexes compared to many other TPP derivatives [80, 83]. The risk of phototoxicity mediated by **Mn‐8** is thus negligible.

### 2.5 In Vivo Dynamic Contrast‐Enhanced MRI Study (DCE‐MRI)

**FIGURE 6:**
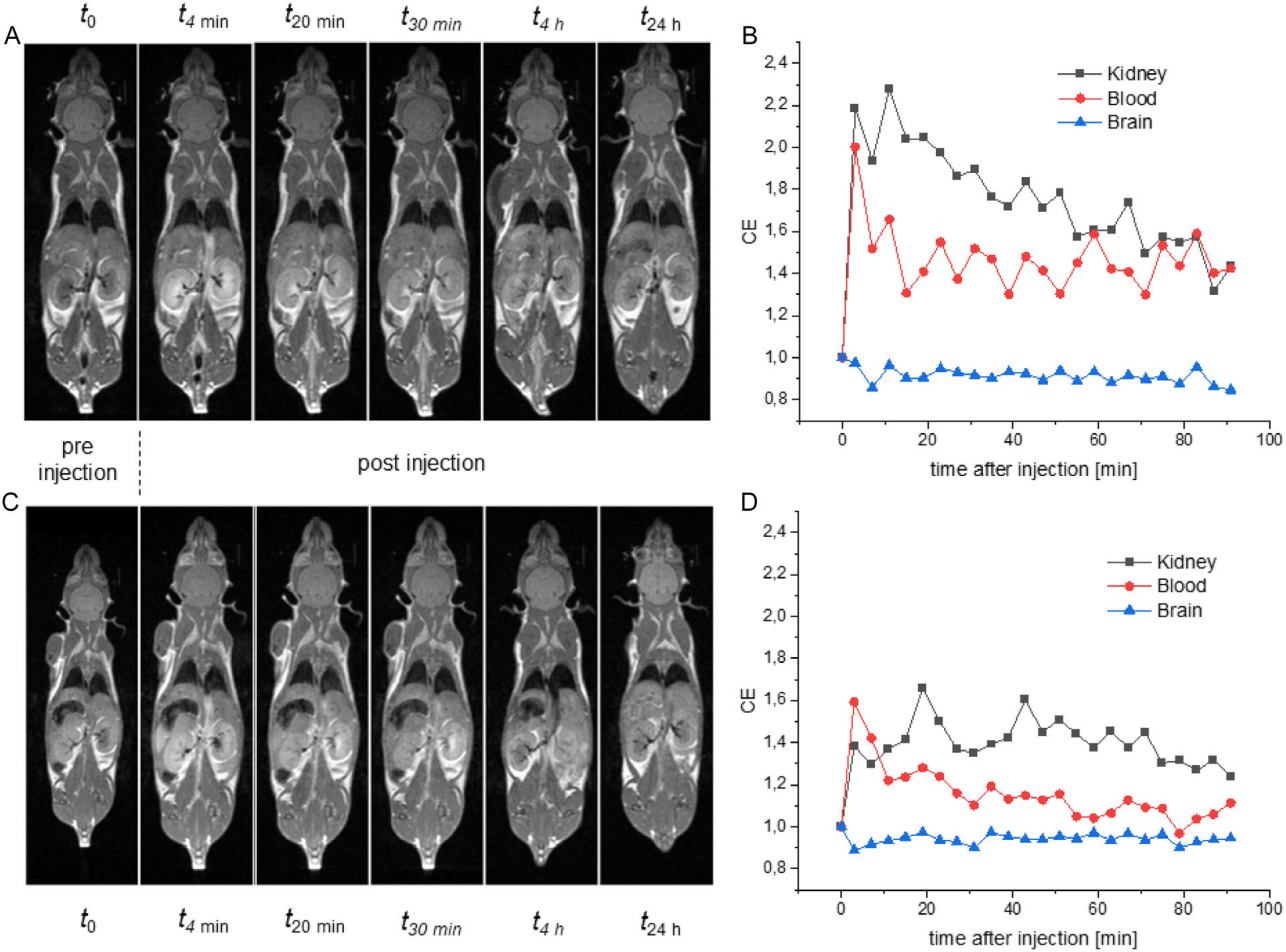
(A) DCE‐MRI following the injection of **Mn‐8** (0.4 mM/kg) into the tail vein of a mouse (sample size *n* = 1). Selected timepoints are shown. (B) Contrast enhancement CE of **Mn‐8** observed for different compartments relative to tissue contrast prior to injection. (C) DCE‐MRI following the injection of gadoteric acid **Gd‐1** (0.4 mM/kg) into the tail vein of a mouse (sample size *n* = 1). Selected timepoints are shown. (D) CE of gadoteric acid **Gd‐1** observed for kidney, blood, and brain relative to tissue contrast prior to injection.

The contrast‐enhancing properties of **Mn‐8** were evaluated in mice (Figure 6)*.* As for control, gadoteric acid **Gd‐1** was injected (dose: 0.4 mM/kg, this corresponds to the human equivalent dose calculated according to a literature procedure [84] assuming a typical human dose of 0.1 mM/kg) using a 0.05 m solution prepared by diluting commercially available Dotarem. **Mn‐8** solution was also injected (dose: 0.4 mM/kg) using a 0.05 m solution into the tail vein of healthy mice. *T*_1_‐weighted MRI measurements were conducted every 4 min following the injection for 90 min, then at 4  and 24 h post injection. The rate of contrast enhancement for different regions of interest was calculated.

Due to the high hydrophilicity of compound, **Mn‐8** contrast enhancement is mainly observed in shallow compartments such as kidney and blood. **Mn‐8** does not appear to transverse the blood‐brain barrier as shown in Figure 6B. Contrast enhancement in brain tissue is not observable, while significant contrast enhancement was observed in kidney and blood. Compared to gadoteric acid, the CE observed for **Mn‐8** at the initial time point 4 min after the injection was increased by 25% in blood and 55% in kidney. **Mn‐8** was well‐tolerated: no weight loss or change in behavior was observed following the application at this dose. *T*_1_ relaxation times measured for the brain and kidneys 24 h after injection indicated low manganese retention in these tissues, with no significant difference compared to the control injected with gadoteric acid **Gd‐1** (Table S2, in the SI).

## 3 Conclusion

GBCAs are the clinical standard for *T*_1_‐weighted MRI. However, concerns about their safety make MBCAs promising alternatives. Herein, the synthesis and characterization of two novel zwitterionic Mn(III) porphyrins, **Mn‐8** and **Mn‐9**, with high aqueous solubility, and remarkable thermodynamic and kinetic stability are reported.

Both zwitterionic complexes have a high *T*_1_ relaxivity, comparable to the clinical high‐relaxivity GBCA gadopiclenol and much higher than gadoteric acid. The high relaxivity of **Mn‐8** and **Mn‐9** can be attributed to their hydrodynamic diameter and thus a relatively high rotational correlation time. The highest relaxivity with *r*_1_ = 14.5 mm^−1^s^−1^ (water, 1.4 T, 37°C) was measured for complex **Mn‐8** with four sulfobetaine groups. The analogue **Mn‐9** with a more compact structure and four *N*‐oxide groups has a slightly lower relaxivity with *r*_1_ = 12.6 mm^−1^s^−1^ under the same conditions. It can be concluded that the decoration of Mn(III)‐porphyrins with zwitterions leads to highly water‐soluble complexes with a low tendency for aggregation and is thus a prerequisite for high relaxivity. However, the study presented is limited with respect to the analysis of the effects of zwitterionic groups on relaxivity and should be complemented by more detailed investigations for example with NMRD measurements in the future. The tetraazido‐substituted TPP **5** is a particularly valuable compound in this context because it allows the rapid variation of groups attached to a common TPP core via click chemistry. A systematic analysis of charge, polarity and molecular size in a comparable set of compounds is therefore feasible.

The incorporation of Mn(III) into the porphyrin core seems to effectively suppress the generation of singlet‐oxygen, thus minimizing the risk for phototoxicity. **Mn‐8** showed no signs of toxicity in a first in vivo experiment and led to strong contrast enhancement in mice, particularly in blood and kidney. In addition, **Mn‐8** appears to not cross the blood–brain barrier. However, a more detailed investigation of in vivo pharmacokinetics and toxicity with more animals is clearly required in future experiments.

In summary, the zwitterionization of Mn‐TPP complexes led to excellent water solubility, high relaxivity, and stability of both zwitterionic complexes **Mn‐8** and **Mn‐9**, which is promising for in vivo applications. In addition, the high hydration of the zwitterions led to a fast clearance and low tissue retention of the complexes in vivo. These features suggest that zwitterionic Mn(III)‐porphyrins warrant further exploration as MR contrast agents [85, 86].

## 4 Experimental Section

### 4.1 General Information

All commercially available reagents and starting materials were purchased from Sigma Aldrich, TCI, abcr or BLDpharm and were used without further purification. Gadopiclenol was purchased as Vueway from Bracco Imaging SpA as a 0.5 m aqueous solution. Mn‐DPDP was purchased from Toronto Research Chemicals Inc. Dem. Water was obtained through purification by PURELAB Classic from ELGA LabWater. Oxygen or moisture‐sensitive reactions were performed under a protective nitrogen atmosphere in flame‐dried glassware. The progress of reactions was monitored by TLC on silica gel ALUGRAM Xtra SIL G/UV254 (normal phase) from Macherey‐Nagel. Detection was achieved via UV (*λ* = 254 and 364 nm). Purification via flash chromatography was performed on the Isolera Prime system from Biotage with self‐packed cartridges from Macherey‐Nagel filled with POLYGOPREP 60–80 silica gel. Reverse phase flash chromatography was performed on the puriFlash450 by Interchim with CHROMABOND Flash RS 25 C18 60 nm or CHROMABOND Flash RS 40 C18 60 nm cartridges from Macherey‐Nagel, eluting with MeCN/H_2_O containing 0.1% formic acid. Compounds were freeze‐dried after dissolving in H_2_O/CH_3_CN using the ALPHA 2–4 LDplus system from Martin Christ. UV/vis measurements were conducted on the GENESYS 10uv Scanning photometer by Thermo Fisher Scientific, utilizing 10 mm quartz cuvettes by Hellma GmbH & Co. KG. HRMS‐ESI‐MS measurements were performed using a 6224 ESI‐TOF spectrometer coupled with a HPLC 1200 series from Agilent Technologies with a Agilent Zorbax Extend C18, 2.1 × 50 mm. Non‐HRMS‐ESI‐MS measurements were performed using a Agilent 1260 Infinity II HPLC coupled to the amaZon SL mass spectrometer from Bruker Daltonics with a Macherey‐Nagel EC Nucleodur C18 Gravity‐SB, 5 µm. NMR spectra were measured with Bruker Avance III HD 600 MHz and Bruker Avance I 400 MHz spectrometers. Chemical shifts (δ) are expressed in parts per millions (ppm).

### 4.2 Relaxivity Measurements at 1.4 T

The longitudinal relaxivity *r*_1_ at 60 MHz (1.41 T) was determined based on spin‐lattice relaxation time *T*_1_ measurements using a Bruker Minispec mq60 analyzer at 37°C. *T*_1_ values were obtained via the standard inversion recovery pulse sequence (180°–*τ*–90°) at 37 ± 0.1°C, sample size *n* = 1. The relaxivity *r*_1_ was calculated by plotting the inverse relaxation time (1/*T*_1_) against the complex concentration and determining the slope and standard error using linear regression analysis in OriginLab 2025. The concentration of the complexes was precisely quantified by ICP‐MS.

The tranverse relaxivity *r*_2_ at 60 MHz (1.41 T) was determined based on Carr‐Purcell‐Meiboom‐Gill echo sequence using a Bruker Minispec mq60 analyzer at 37°C. *T*_2_ values were obtained *via* the standard inversion recovery pulse sequence (180°–*τ*–90°) at 37 ± 0.1°C, sample size *n* = 1. The relaxivity *r*_2_ was calculated by plotting the inverse relaxation time (1/*T*_2_) against the complex concentration and determining the slope and standard error using linear regression analysis in OriginLab 2025. The concentration of the complexes was precisely quantified by ICP‐MS.

### 4.3 ICP‐MS

Manganese concentrations were determined by ICP‐MS. Prior to ICP‐MS measurements, 10 µL of the samples were digested in 1 mL of nitric acid (ROTIPURAN Supra, 69%) for 24 h at room temperature in pre‐cleaned tubes. Afterwards, all samples were filled up to 12 mL with ultrapure water. The digested samples were then diluted 1:100 prior to analysis. The measurements were performed on an Agilent Technologies 7800x ICP‐MS (Agilent Technologies Inc., Santa Clara, USA) equipped with a quadrupole mass analyzer. Prior to measurement, the ICP‐MS setup was tuned with a mixture of Ce, Co, Li, Tl, and Y at a concentration of 1 ppb (Agilent Technologies Inc., Santa Clara, USA). External calibration was performed using mixed element standards purchased from Merck KGaAA and PerkinElmer Inc. Calibration solutions containing Mn at concentrations of 0–1000 ppb were prepared freshly. Quantitation was performed by external calibration, corrected by internal standard (72 Ge for Mn). To ensure stability during the measurement a quality control sample containing 100 ppb Mn was measured.

### 4.4 Kinetic Inertness in Acidic Media (Method I)

Kinetic inertness in acidic media was evaluated *via* relative UV/vis measurements of the Mn(III)‐porphyrin soret band absorbance at 467 nm. Porphyrins **Mn‐8** and **Mn‐9** were dissolved in diluted aq. HCl at pH 3.1 or pH 1.2 without buffer addition and the pH of the resulting solutions was verified. For each porphyrin, 25 mL of 8 µm solution at either pH 3.1 and pH 1.2 was prepared. The solutions were incubated at 37°C, a 1 mL aliquot was withdrawn immediately, and the soret band absorption was measured to define *t* = 0. Sampling was conducted at days 0, 1, 2, 5, 10, and 15.

### 4.5 Kinetic Inertness in Acidic Media (Method II)

Porphyrins **Mn‐8** and **Mn‐9** were dissolved in dilute HCl at pH 1.2 (1.25 mm), and the spin lattice relaxation time *T*_1_ was measured immediately to define the timepoint *t* = 0 min. The mixtures were then incubated at 37°C, and *T*_1_ was recorded at 3 h, 1, 2, 3, 7, and 15 days. All *T*_1_ measurements were performed 37°C +/‐ 0.1°C using a Bruker Minispec mq60 analyser employing the standard inversion recovery pulse sequence. The *T*_1_ values were subsequently plotted as a ratio relative to the initial value measured at *t* = 0 min.

### 4.6 Inertness Against 1000 Fold Zn(II) Excess (Method I)

Inertness against excess Zn(II) was evaluated via UV/vis measurements of the Mn(III)‐porphyrin soret band absorbance at 467 nm relative to the Zn(II)‐porphyrin soret band absorbance at 432 nm. Mn(III)‐porphyrins were dissolved in MilliQ water at concentrations of 8 µm containing 8 mm ZnCl_2_. For each porphyrin, 25 mL of solution was prepared. The solutions were incubated at 37°C, a 1 mL aliquot was withdrawn immediately, and the absorption spectrum was measured to define *t* = 0. The ratio of soret band absorbance of Mn(III) porphyrins against soret band absorbance of Zn(II) porphyrins was plotted for each sampling point. Sampling was conducted at days 0, 1, 2, 5, 10, and 14. Porphyrins **Zn‐8** and **Zn‐9** were used as spectral reference.

### 4.7 Inertness Against Zn(II) Transmetalation (Method II)

Prewarmed to 37°C, solutions of **Mn‐8** and **Mn‐9** (2.5 mm) and ZnCl_2_ (2.5 mm) in aqueous 1X PBS buffer (pH 7.4) were mixed in a 1:1 ratio, and the spin lattice relaxation time *T*_1_ was measured immediately to define the timepoint *t* = 0 min. The mixtures were then incubated at 37°C and *T*_1_ was recorded at 3 h, 1, 2, 3, 7, and 15 d. All *T*_1_ measurements were performed 37°C +/− 0.1°C using a Bruker Minispec mq60 analyser employing the standard inversion recovery pulse sequence. The *T*_1_ values were subsequently plotted as a ratio relative to the initial value measured at *t* = 0 min.

### 4.8 Inertness Against Reductive Species

Porphyrins **Mn‐8** and **Mn‐9** were incubated at concentrations of 40 µM in 1 mL of de‐aerated 1X PBS buffer (pH 7.4) containing 4 mM glutathione, at 37°C in sealed quartz cuvettes, under argon atmosphere to prevent re‐oxidation due to atmospheric oxygen. Immediately after addition of porphyrins, the UV/vis spectrum of each porphyrin was analyzed. Measurements were repeated after 24 h of incubation, and the spectral data compared.

### 4.9 Inertness in Human Serum

Porphyrins **Mn‐8** and **Mn‐9** were incubated at concentrations of 10 µm in 3 mL of human serum (Capricorn Scientific, male, Type AB, Cat. No. HUM‐3B) supplemented with 0.02% sodium azide at 37°C. Immediately after addition of porphyrins to serum, a 1 mL sample was drawn from each solution and filtered through an Amicon filter unit (30 kDA MWCO by Merck Millipore) with the aid of centrifugation (20 min, 50.000 rpm). The resulting filtrate was analyzed via LC‐MS utilizing the gradient listed in Table S1. Measurements were repeated 21 d following addition. The integrated peak areas at 450 nm were compared to day 0 of incubation. LC/MS data are depicted as Figures S28−31.

The human serum was obtained commercially from Capricorn Scientific, Cat. No. HUM‐3B. According to the supplier, all samples were collected from healthy donors in FDA‐licensed facilities in the USA, after written informed consent, in compliance with relevant national regulations and approved by an independent ethics committee. The samples were fully anonymized prior to purchase.

### 4.10 Generation of Singlet Oxygen

Generation of singlet oxygen was evaluated via a previously established DPBF‐based essay. Known photosensitizer rose bengal and porphyrins **5** and **Mn‐8** were dissolved in DMF at concentrations of 4 µm containing 40 µm DPBF. The resulting solutions were handled under strict light exclusion. The solutions were transferred into 1 × 1 cm quartz cuvettes and placed inside a light proof container containing a white‐light LED placed exactly 100 mm from the cuvette. The relative power output at this distance was determined to be 0.50 mW at 467 nm. The solutions were irradiated, and the absorbance of DPBF at 415 nm was measured at 0, 1, 2, 3, 4, 5, 6, 7, 8, 9, and 10 min of irradiation. The decrease of absorbance relative to *t*_0_ was plotted against irradiation time. A negative control containing solely DPBF without any photosensitizer was used to quantize the autodegradation of DPBF under irradiation. Measurements were performed as triplicates. Mean values and standard deviation were plotted in OriginLab 2025. The spectral data of the utilized LED is given at the end of the supporting information (Figure S26 and S27).

### 4.11 HPLC Analysis

(HPLC) high‐performance liquid chromatography analysis was conducted using an Agilent 1260 Infinity II HPLC from Agilent Technologies Inc. equipped with a Macherey‐Nagel EC Nucleodur C18 Gravity‐SB, 5 µm, 150 × 3 mm running the gradient program listed in Table S1. Detection was performed via the integrated DAD at 254 nm.

### 4.12 In Vivo DCE‐MRI and Manganese Retention Assessment

All animal experiments were approved by and conducted under the guidelines of Université Laval and Centre de recherche du Centre hospitalier universitaire de Que'bec's animal ethical committee (project CHU‐23−1340), under regulations of the Canadian Council on Animal Care. 6‐week‐old BALB/c female mice (Charles River, Montreal, Canada) were used for the study. Mice were first anaesthetized with 3% isoflurane in an induction box and transferred to the MRI mouse bed while kept under anesthesia by means of a nose cone integrated into the bed. The animals were continuously monitored for respiration with a small animal monitoring and gating system (model 1025T; SA Instruments, Stony Brook, NY). The mice were cannulated in previously dilated caudal tail vein (30 G, winged needle), connected to a catheter (280 μm ID intramedic polyethylene tubing PE‐10, 130 cm, total volume = 80 μL) prewashed with heparin, and connected to the contrast media syringe. The needle was secured with adhesive (3 M Vetbond) and protective ointment (Lacri‐Lube) was applied on the mice's eyes. Then, the animals were inserted in a 35‐mm‐diameter RF coil and scanned using a 1T small‐animal MRI system (M2M, Aspect Imaging, Israel). The mice were scanned using a *T*_1_‐weighted 2D spin−echo sequence in coronal orientation. The scanning parameters were as follows: TR: 700 ms; TE: 14 ms; slice thickness: 0.8 mm; slice gap: 0.1 mm; field‐of‐view = 100 × 100 mm; dwell time = 16 μs; fα = 90°; encoding: 340/200; 1 excitation; total duration = 4 min 16 s. Two pre‐injection images were acquired as references (S_0_).

Two animals were injected with 140 µL of a 0.042 m gadoteric acid **Gd‐1** solution prepared by diluting Dotarem 1:12 in saline solution. One animal was injected with 150 µL of a 0.05 m **Mn‐8** solution. This dose was prepared to provide equivalent longitudinal relaxation time as injected **Gd‐1** solution used as control (*T*_1_ = 9,577 ± 0,002 ms). MRI imaging was conducted every approximately 4 min following the injection for 90 min, then at 4  and 24 h post injection.

## Supporting Information

Additional supporting information can be found online in the Supporting Information section.

## Funding

This study was supported by Fraunhofer‐Gesellschaft (178–600040).

## Conflicts of Interest

The authors declare no conflicts of interest.

## Supporting information

Supplementary Material

## Data Availability

The data that supports the findings of this study are available in the supplementary material of this article.
