# Supplementary material for: Zwitterionic Mn(III)‐Tetraphenyl Porphyrins: Water‐Soluble MRI Contrast Agents with High Relaxivity
Source: ChemMedChem. 2026 Apr 16;21(8):e70268. doi: 10.1002/cmdc.70268 (PMC13084297; doi:10.1002/cmdc.70268)
Supplement: Supplementary file 1 — Supplementary Material [file CMDC-21-e70268-s001.pdf]

Supporting Information

Zwitterionic Mn(III)-Tetraphenyl Porphyrins: Water-Soluble MRI-Contrast Agents With High-Relaxivity

Darius Ludolfs,[a] Lennart F. V. Spickschen,[a] Stefanie Bredehöft,[a] Verena R. Schulze,[b] Marie Oest,[c] Samila Leon Chaviano,[d] Neus Feliu,[b] Markus Fischer,[c] Marc-André Fortin,[d] John V. Frangioni[e] and Wolfgang Maison*[a]

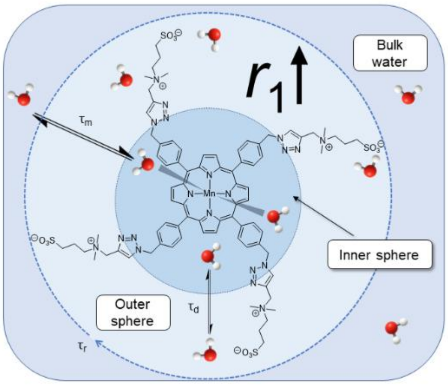

[a] D. Ludolfs, L. Spickschen, Stefanie Bredehöft, Prof. Dr. W. Maison
Department of Chemistry
Institute of Pharmacy
Universität Hamburg, Bundesstrasse 45, 20146 Hamburg, Germany
E-mail: wolfgang.maison@uni-hamburg.de

[b] V. R. Schulze, Dr. N. Feliu
Fraunhofer Institute of Applied Polymer Research IAP
Center of Applied Nanotechnology CAN
Grindelallee 117, 20146 Hamburg, Germany

[c] M. Oest, Prof. Dr. M. Fischer
Hamburg School of Food Science
Institute of Food Chemistry
Universität Hamburg, Grindelallee 117, 20146 Hamburg, Germany

[d] Prof. Dr. M.-A. Fortin, S. Leon Chaviano
Axe Oncologie, Centre de Recherche du CHU de Québec – Université Laval, 2705, boul. Laurier, Québec, QC, G1V4G2, Canada; Centre de Recherche sur le Cancer (CRC) de l'Université Laval, 9 Rue McMahon, Québec, QC, G1R 3S3, Canada; Département de Génie des Mines, de la Métallurgie et des Matériaux, Université Laval, Québec, QC, G1V 0A6, Canada.

[e] Dr. J. V. Frangioni
Curadel Pharma
28120 Hunters Ridge Blvd, Suites 6-7, Bonita Springs, FL 34135, USA

WILEY-VCH

Relaxivity Measurements at 1.4 T

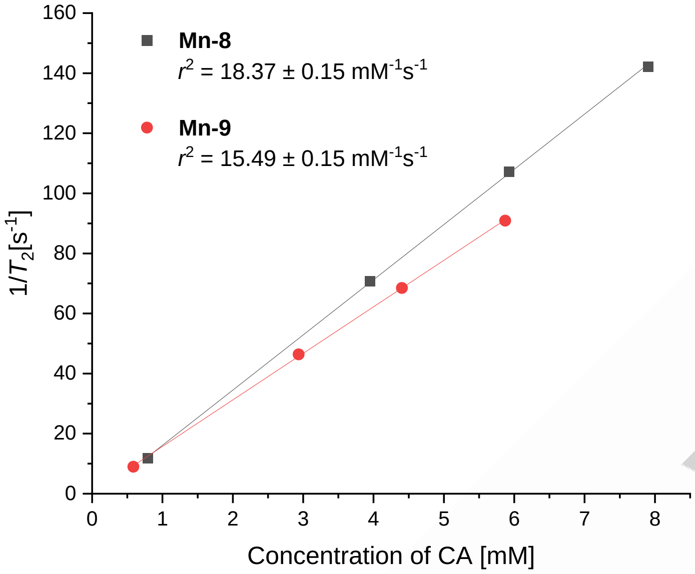

Figure S1. Calculation of transverse relaxivity r_2 of compounds **Mn-8** and **Mn-9**

HPLC analysis

Table S1: HPLC elution gradient

Time	% H_2O (0.1% FA)	% MeCN (0.1% FA)	Flow rate (mL/min)
0-2 min	98	2	0.2
2-20 min	2	98	0.2
20-23 min	2	98	0.2
23-25 min	98	2	0.2
25-30 min	98	2	0.2

In vivo DCE-MRI and manganese retention assessment

Regions of interest (ROI) were drawn on MRI images (kidney cortex, kidney pelvis, abdominal aorta, brain) using the 3D Slicer imaging software (5.9.0 version). Mean signal values were calculated ($S_{kidney\ cortex}$, $S_{kidney\ pelvis}$, $S_{abdominal}$ S_{aorta}, S_{brain}, respectively) and compared with the mean signal values from adjacent air volumes (S_{air}). Contrast ratios were calculated as follows:

$$Contrast\ ratio\ (CR) = \frac{S_{organ} - S_{air}}{S_{muscle} - S_{air}}$$

Formula S1: Calculation of contrast ratio *CR.*

WILEY-VCH

Contrast enhancement at time points was calculated as follows:

$$Contrast\ enhancement\ (CE) = \frac{CR_{t0}}{CR_{tx}}$$

Formula S2: Calculation of contrast enhancement *CE.*

Where CR_{t0} and CR_{tx} are contrast rations at times t_0 (prior to injection) and t_x.

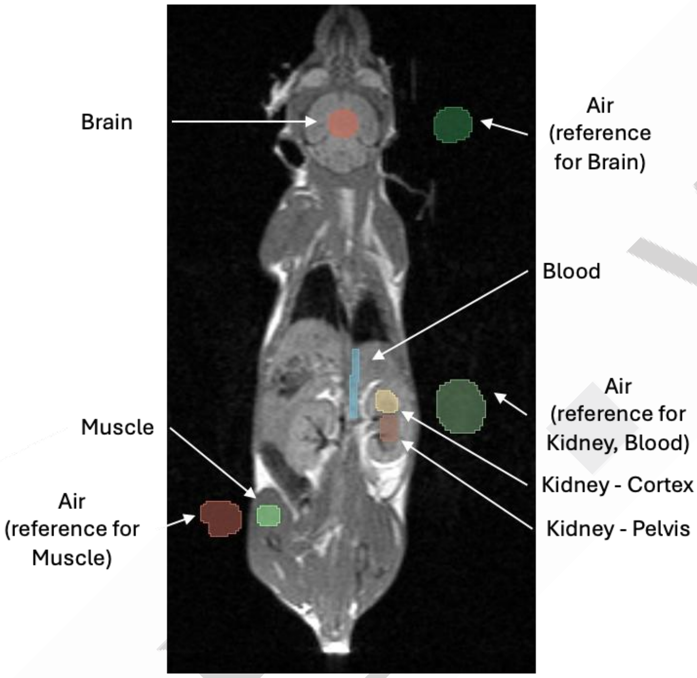

Figure S2: Regions of interest used for the calculation of contrast enhancement ratios.

Also, manganese retention was assessed in brain and kidneys by relaxometric measurements at 24 h post injection. A non-injected animal was used as control. Organs were harvested after MRI scan performed at 24 hours post injection, then weighed and inserted into an NMR tube (borosilicate glass, OD: 7.5 mm, ID: 6 mm, L: 18 cm). Longitudinal relaxation times T_1 were measured using an NMR relaxometer (Bruker Minispec 60 mq, 60 MHz, 1.41 T, 37 °C).

WILEY - VCH

Table S2: Results of Mn retention assessment by relaxometry.

	Control (non injected animal) (n=1)		**Gd-1** (n=2)		**Mn-8** (n=1)	
	Brain	Kidneys	Brain	Kidneys	Brain	Kidneys
Average weight (g)	0.324	0.315	0.255	0.315	0.324	0.327
Mean T_1 (ms)	**853**	**677**	**838**	**585**	**902**	**570**
STD	3	2	6,7	3,6	1	5
Percentage deviation from control	N/A	N/A	-1.8%	-13.6%	5.7%	-15.8%

Synthetic protocols:

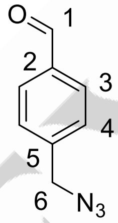

4-azidomethylbenzaldehyde (4):
4-Bromomethylbenzaldehyde (943 mg, 4.74 mmol, 1.00 eq.) and potassium carbonate (2.61 g, 18.9 mmol, 4.00 eq.) were dissolved in 20 mL DMF. Sodium azide (466 mg, 7.16 mmol, 1.51 eq.) was added and the yellow suspension was heated at 60 °C for 1 h under vigorous stirring. After complete conversion of the starting material (TLC) the mixture was allowed to cool to room temperature und poured into 500 mL of MilliQ water. The mixture was extracted with 3 x 50 mL ethyl acetate and the combined organic phases were washed with 2 x 100 mL MilliQ water, once with 100 mL brine and dried over sodium sulfate. Removal of the solvent *in vacuo* gave 4-azidomethylbenzaldehyde as a colorless oil (734 mg, 4.56 mmol, 96% yield). The product was used in the next reaction step without further purification. ^{1}H NMR (400 MHz, $CDCl_3$) δ [ppm] = 10.00 (s, 1H, H-1), 7.88 (d, J = 8.2 Hz, 1H, H-3), 7.47 (d, J = 8.1 Hz, 2H, H-4), 4.44 (s, 2H, H-6).^{13}C-NMR (151 MHz, $CDCl_3$): δ [ppm] = 191.8 (C-1), 141.7 (C-5), 136.2 (C-2), 130.3 (C-3), 128.6 (C-4), 54.3 (C-6).

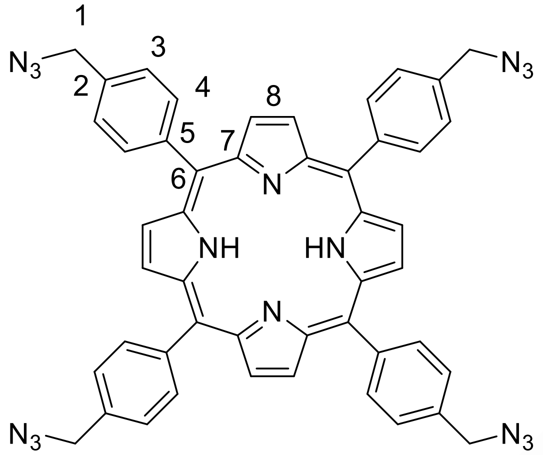

5,10,15,20-Tetrakis(4-azidomethylphenyl)porphyrin (5):
In a flame dried 500 mL round bottom flask freshly distilled pyrrole (0.2 mL, 2.91 mmol, 1.00 eq.) and 4-azidomethylbenzyladehyde (469 mg, 2.91 mmol, 1.00 eq.) were dissolved in 300 mL of anhydrous CH_2Cl_2 and purged with dry nitrogen. $BF_3{\cdot}Et_2O$ in CH_2Cl_2 (0.37 mL, 0.789 M, 0.29 mmol, 0.1 eq.) was added and the solution was stirred at room temperature overnight. DDQ (654 mg, 2.87 mmol, 1.00 eq.) was added and the deep purple solution was further stirred for one hour. Et_3N (0.41 mL, 2.96 mmol, 1.00 eq.) was added and the black solution was filtrated over silica gel, eluting with CH_2Cl_2. The solvent was removed *in vacuo* and the black residue was further purified *via* flash chromatography (silica gel, CH_2Cl_2) to yield the compound as a deep violet solid (132 mg, 0.16 mmol, 5%). Procedure adapted from *Le Pleux* et al.[1]
^{1}H NMR (500 MHz, $CDCl_3$) δ [ppm] = 8.85 (s, 8H, H-8), 8.24 (d, J = 7.8 Hz, 8H, H-4), 7.70 (d, J = 7.8 Hz, 8H, H-3), 4.72 (s, 8H, H-1), -2.76 (s, 2H, N-H). ^{13}C-NMR (101 MHz, $CDCl_3$) δ [ppm] = 142.2 (C-7), 135.2 (C-6), 135.0 (C-3, C-4), 126.7 (C-2, C-5), 119.7 (C-8), 55.0 (C-1) UV/vis (DMF) λmax, nm (ε, L · mol^{-1} · cm^{-1}): 419 (349586), 514 (13669), 549 (6459), 591 (3755), 649 (3555).

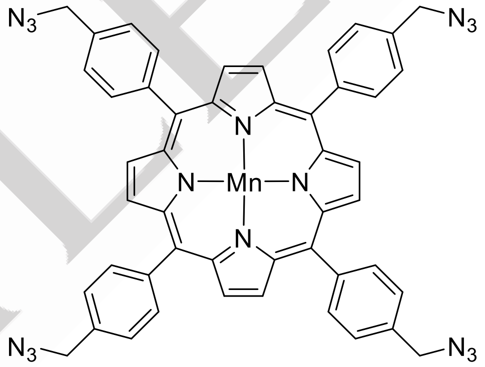

Mn(III)-5,10,15,20-Tetrakis(4-azidomethylphenyl)porphyrin (Mn-5), method i):
To a solution of **5** (80 mg, 96 µmol, 1 eq.) in 20 mL DMF and 0.1 mL DIPEA was added manganese(II)chloride tetrahydrate (95 mg, 0.49 mmol, 5 eq.). The suspension was purged with dry nitrogen for 30 minutes and heated to 130 °C for 1 h under strong stirring. After complete conversion of the starting material (TLC) the solution was allowed to cool to room temperature and the solvent was removed under reduced pressure. The dark green residue was suspended in 30 mL of CH_2Cl_2 and washed with 3 x 100 mL MilliQ water. The solvent was removed under reduced pressure and the product was further purified by flash chromatography (silica gel, CH_2Cl_2). The product was obtained as a deep green solid (88.1 mg, 0.095 mmol, 99%). HRMS (ESI) *m/z* $[M+H]^{2+}$ calcd. for $C_{48}H_{33}MnN_{16}^{2+}$: 444.1222, found: 444.1219. t_R (C18 Gravity-SB): 23.9 min. UV/vis (DMF) λmax, nm (ε, L · mol^{-1} · cm^{-1}): 380 (35153), 400 (37252), 418 (32533), 468 (83431), 570 (8456), 608 (7115).

WILEY-VCH

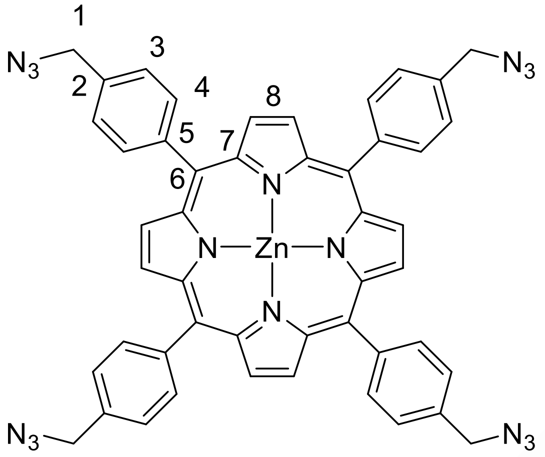

Zn(II)-5,10,15,20-Tetrakis(4-azidomethylphenyl)porphyrin (Zn-5), method ii):
To a solution of **5** (80 mg, 96 µmol, 1 eq.) in a mixture of 20 mL CH_2Cl_2 and 20 mL MeOH was added anhydrous zinc(II) acetate (88 mg, 0.48 mmol, 5 eq.) and the suspension was gently refluxed for 3 h. After complete conversion of the starting material (TLC) the mixture was allowed to cool to room temperature and the solvent was removed under reduced pressure. The crude product was purified *via* flash chromatography (silica gel, CH_2Cl_2/ MeOH 97:3) to yield a purple solid as the final product (81 mg, 0.090 mmol, 92% yield). ^{1}H NMR (400 MHz, DMSO-d6) δ 8.78 (s, 8H, H-8), 8.19 (d, J = 7.5 Hz, 8H, H-4), 7.75 (d, J = 5.1 Hz, 8H, H-3), 4.83 (s, 2H, H-1). ^{13}C-NMR (101 MHz, DMSO-d6) δ[ppm] = 149.75 (C-7), 143.06 (C-5), 135.51 (C-4), 134.42 (C-8), 131.62 (C-2), 127.10 (C-3), 119.92 (C-6), 53.68 (C-1) HRMS (ESI) *m*/*z* [M+H]$^+$ calcd. for $C_{48}H_{33}N_{16}O_{12}Zn^+$: 897.2366, found: 897.2357 UV/vis (DMF) λmax, nm (ε, L · mol^{-1} · cm^{-1}): 405 (16068), 423 (32932), 519 (1017), 559 (6747), 599 (3109)

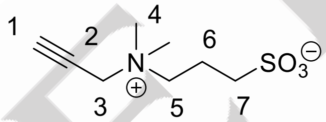

3-(dimethyl(prop-2-ynyl)ammonio)propan-1-sulfonate (6)
To a solution of 3-dimethylamino-1-propyne (3.60 mL, 36.0 mmol, 1.00 eq.) in toluene (150 mL) was added 1,3-propanesultone (9.6 g, 6.9 mL, 2.20 eq.) and the mixture was stirred for 18 h at room temperature. The colourless precipitate was isolated via filtration, washed with toluene (3 x 15 mL), acetone (3 x 15 mL) and dried under high vacuum. 3-(dimethyl(prop-2-ynyl)ammonio)propan-1-sulfonate (5.41 g, 26.3 mmol, 73%) was isolated as a colourless solid. Procedure adapted from *Niu* et al.[2] ^{1}H-NMR ($CDCl_3$, 600 MHz) δ = 4.32 (2H, d, $^4J_{H,H}$ = 2.6 Hz, H-3), 3.64 (2H, m, H-7), 3.31 (1H, t, $^4J_{H,H}$ = 2.5 Hz, H-1), 3.24 (6H, s, H-4), 3.02 (2H, t, $^3J_{H,H}$ = 7.2 Hz, H-5), 2.28 (2H, m, H-6) HRMS (ESI) *m*/*z* [M+H]$^+$ calcd. for $C_8H_{16}NO_3S^+$: 206.0846, found: 206.0848

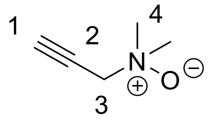

3-dimethylamino-1-propyne *N*-oxide (7)
Under nitrogen atmosphere a solution of 3-dimethylamino-1-propyne (2.00 g, 24.0 mmol, 1.00 eq.) in dry CH_2Cl_2 (24 mL) was cooled to 0 °C in an ice bath and a solution of *m*CPBA (75% with H_2O, 5.54 g, 24.0 mmol, 1.00 eq) in dry CH_2Cl_2 (24 mL) was added in a single portion. The reaction mixture was warmed to room temperature and stirred for 3 h. Afterwards the reaction mixture was passed through a column of alkaline alumina (15 times the weight of the combined starting material, 110 g). The column was first eluted with CH_2Cl_2 and then with CH_2Cl_2 / MeOH 95:5 to elute the product. All volatiles were removed *in vacuo* (bath temperature at room temperature). The product was dried under high vacuum to yield 3-dimethylamino-1-propyne *N*-oxide (1.98 g, 83%) as a colourless solid. Procedure adapted from *Galan* et al.[3] ^{1}H-NMR (D_2O, 300 MHz) δ = 4.20 (s, 2H, H-3), 3.35 (s, 1H, H-1), 3.30 (s, 6H, H-4) HRMS (ESI) *m*/*z* [M+H]$^+$ calcd. for $C_5H_{10}NO^+$: 100.0757, found: 100.0761

WILEY-VCH

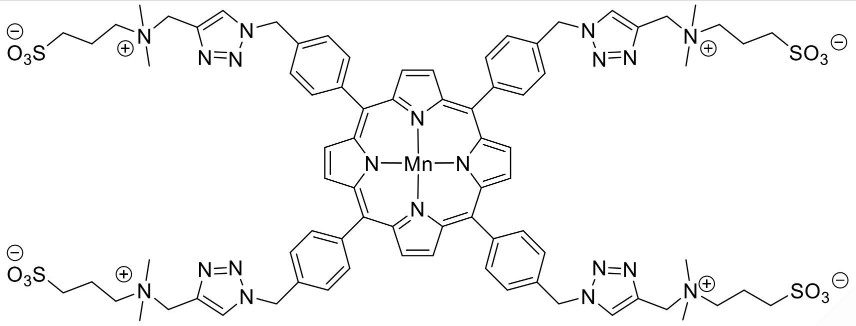

Mn-8

To 50 mg of **Mn-5** (56 µmol, 1eq.) in 10 mL of DMF/ H_2O/ *t*BuOH (2:1:1) were added 139 mg of **6** (0.67 mmol, 12 eq.). Nitrogen gas was bubbled through this dark green solution and copper(I) iodide (1.1 mg, 10 mol%), TBTA (3 mg, 10 mol%) and sodium ascorbate (1.1 mg, 10 mol%) were added. The mixture was stirred at 50 °C under N_2 atmosphere for 1 h. All volatiles were removed under reduced pressure and the dark green residue was purified by flash chromatography (C-18 silica gel, MeCN/ H_2O + 1% formic acid). Residual copper was removed by stirring an aqueous solution of the product over 200 mg QuadraPure TU® for 48 h. Lyophilization yielded the final product as a dark green, voluminous solid (82 mg, 48 µmol, 82%). HRMS (ESI) *m*/*z* $[M+H]^{2+}$ calcd. for $C_{80}H_{93}MnN_{20}O_{12}S_4^{2+}$: 854.2767, found: 854.2763. t_R (C18 Gravity-SB): 14.0 min. UV/vis (H_2O) λmax, nm (ε, L · mol^{-1} · cm^{-1}): 379 (3916), 400 (3874), 467 (6226), 564 (814), 599 (635)

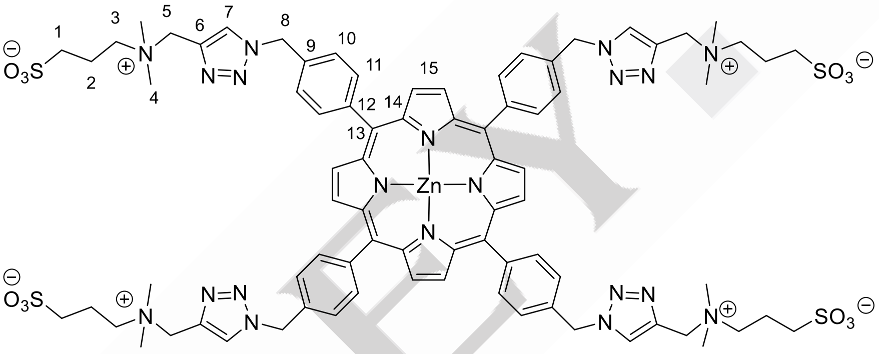

Zn-8

To 50 mg of **Zn-5** (56 µmol, 1eq.) in 10 mL of DMF/ H_2O/ *t*BuOH (2:1:1) were added 139 mg of **6** (0.67 mmol, 12 eq.). Nitrogen gas was bubbled through this dark violet solution and copper(I) iodide (1.1 mg, 10 mol%), TBTA (3 mg, 10 mol%) and sodium ascorbate (1.1 mg, 10 mol%) were added. The mixture was stirred at 50°C under N_2 atmosphere for 1 h. All volatiles were removed under reduced pressure and the violet residue was purified by flash chromatography (C-18 silica gel, MeCN / H_2O + 1% formic acid). Residual copper was removed by stirring an aqueous solution of the product over 200 mg QuadraPure TU® for 48 h. Lyophilization gave the final product as a dark violet, voluminous solid (87 mg, 51 µmol, 91% yield). ^{1}H NMR (400 MHz, DMSO-d6) δ 8.84 (s, 4H, H-7), 8.74 (s, 8H, H-15), 8.18 (d, J = 7.7 Hz, 8H, H-10), 7.73 (d, J = 7.9 Hz, 8H, H-11), 6.04 (s, 8H, H-8), 4.77 (s, 8H, H-5), 3.50 – 3.42 (t, 8H, H-1), 3.34 (m, 35H, H-3, H-4), 2.24 – 2.09 (m, 8H, H-2). t_R (C18 Gravity-SB): 14.1 min. HRMS (ESI) *m*/*z* $[M+H]^+$ calcd. for $C_{80}H_{93}N_{20}O_{12}S_4Zn^+$: 1717.5451, found: 1717.5450 UV/vis (H_2O) λmax, nm (ε, L · mol^{-1} · cm^{-1}): 432 (5904), 560 (408), 600 (209)

WILEY-VCH

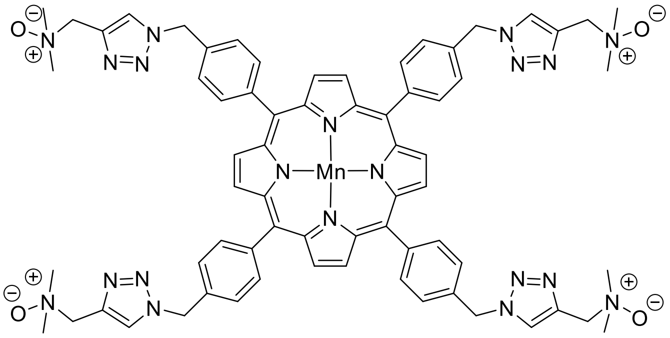

Mn-9

To 50 mg of **Mn-5** (56 µmol, 1eq.) in 10 mL of DMF / H_2O / *t*BuOH (2:1:1) were added 67 mg of **7** (0.67mmol, 12 eq.). Nitrogen gas was bubbled through this dark green solution and copper(I) iodide (1.1 mg, 10 mol%), TBTA (3 mg, 10 mol%) and sodium ascorbate (1.1 mg, 10 mol%) were added. The mixture was stirred at 50°C under N_2 atmosphere for 1 h. All volatiles were removed under reduced pressure and the dark green residue was purified by flash chromatography (C-18 silica gel, MeCN / H_2O + 1% formic acid). Residual copper was removed by stirring an aqueous solution of the product over 200 mg QuadraPure TU® for 48 h. Lyophilization gave the final product as a dark green, voluminous solid (60 mg, 47 µmol, 82% yield). HRMS (ESI) *m/z* $[M+H]^{2+}$ calcd. for $C_{68}H_{69}MnN_{20}O_4^{2+}$: 642.2590, found: 642.2593. t_R (C18 Gravity-SB): 9.8 min. UV/vis (H_2O) λmax, nm (ε, L · mol^{-1} · cm^{-1}): 379 (3050), 400 (3002), 467 (4761), 564 (627), 599 (494)

WILEY-VCH

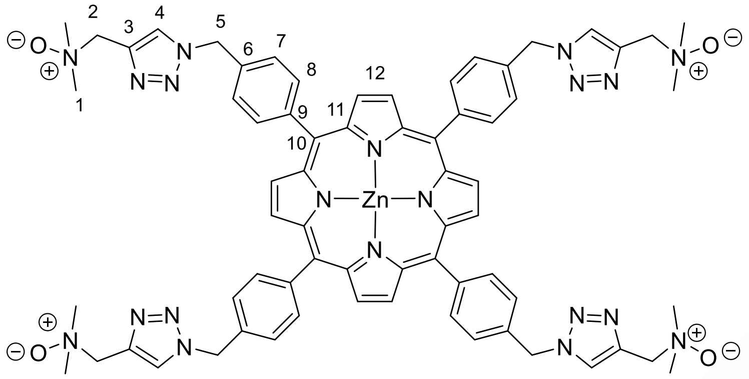

Zn-9

To 50 mg of **Zn-5** (0.056 mmol, 1eq.) in 10 mL of DMF/ H_2O/ *t*BuOH (2:1:1) were added 67 mg of **7** (0.674 mmol, 12 eq.). Nitrogen gas was bubbled through this dark green solution and copper(I) iodide (1.1 mg, 10 mol%), TBTA (3 mg, 10 mol%) and Na-ascorbate (1.1 mg, 10 mol%) were added. The mixture was stirred at 50°C under N_2 atmosphere for 1 h. All volatiles were removed under reduced pressure and the dark green residue was purified by flash chromatography (C-18 silica gel, MeCN + 0.1% formic acid / H_2O + 0.1% formic acid). Residual copper was removed by stirring an aqueous solution of the product over 200 mg QuadraPure TU® for 48 h. Lyophilization gave the final product as a dark green, voluminous solid (66 mg, 0.51 µmol, 91% yield). The ^{1}H-NMR showed broad peaks. ^{1}H NMR (500 MHz, DMSO-d6) δ [ppm] = 9.03 (s, 8H, H-12), 8.64 (s, 4H, H-4), 8.47 (d, *J* = 7.4 Hz, 8H, H-8), 7.96 (d, *J* = 7.7 Hz, 8H, H-7), 6.26 (s, 8H, H-5), 3.87 (m, 34H, H-1, H-2). HRMS (ESI) *m/z* [M+H]$^+$ calcd. for $C_{68}H_{69}ZnN_{20}O_4^+$: 1293.5097, found: 1293.5076. t_R (C18 Gravity-SB): 13.2 min. UV/vis (H_2O) λmax, nm (ε, L · mol^{-1} · cm^{-1}): 432 (3895), 559 (223), 600 (100)

WILEY-VCH

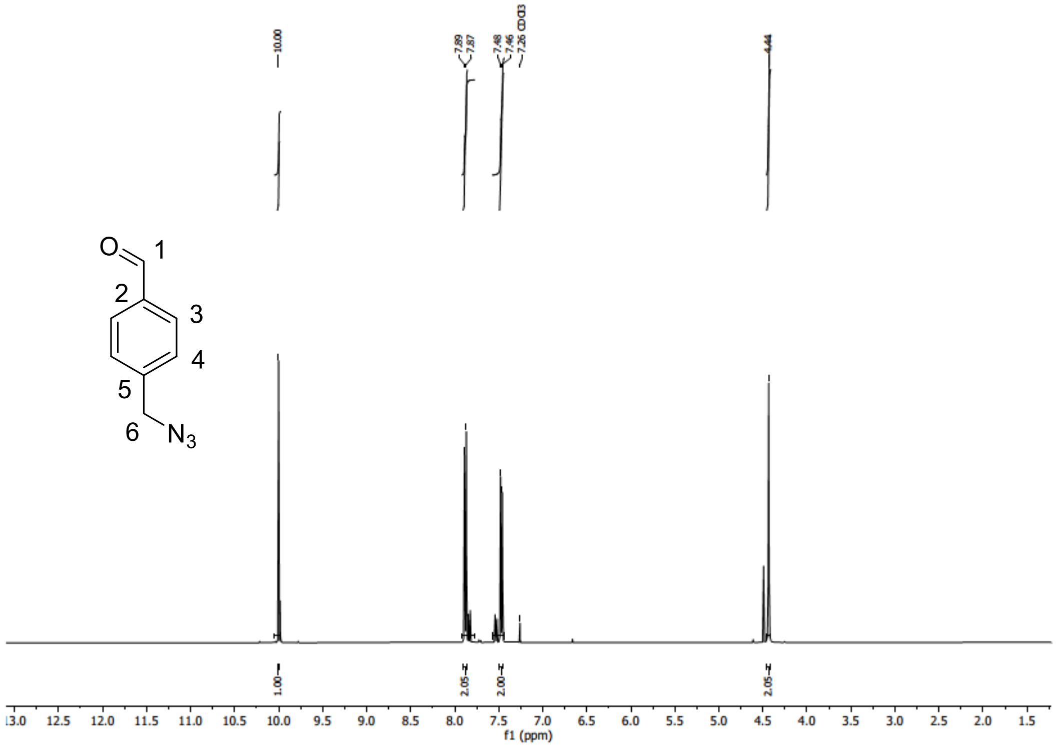

Figure S3. ^{1}H NMR spectrum of compound **4** in $CDCl_3$.

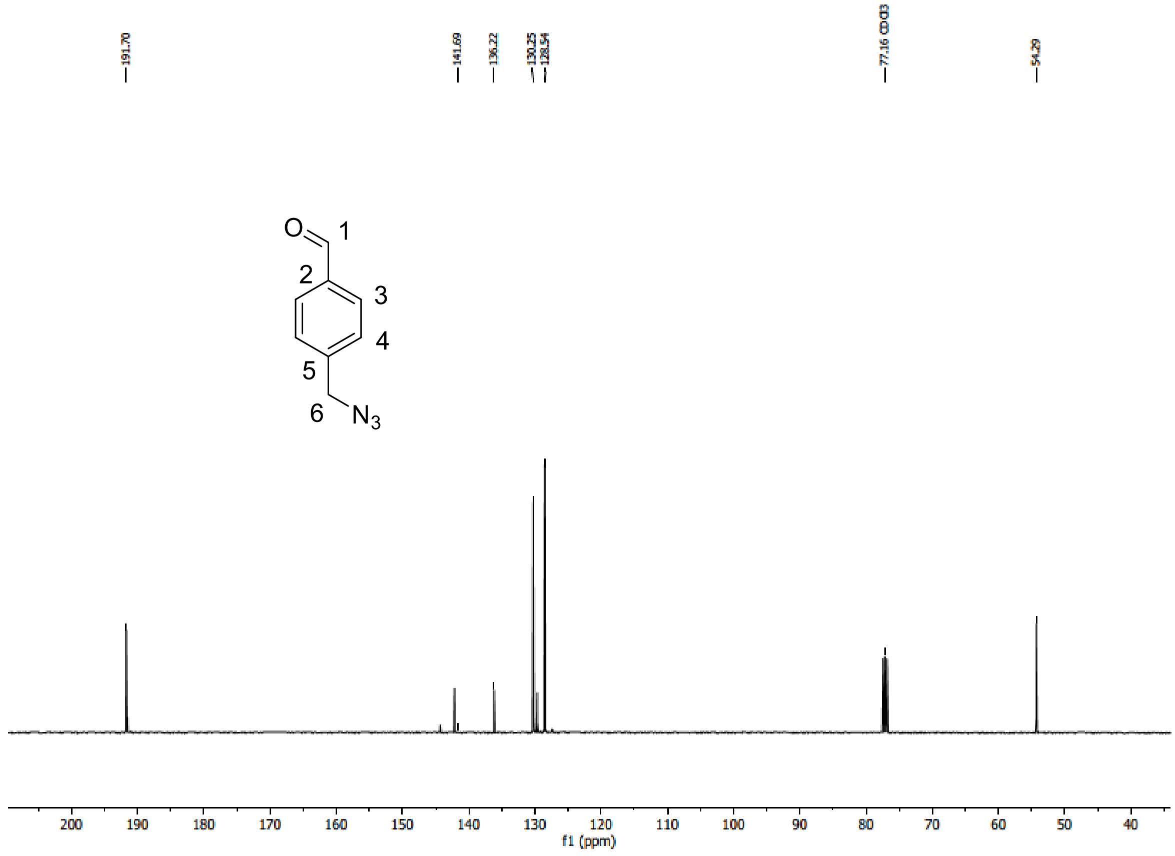

Figure S4. ^{13}C NMR spectrum of compound **4** in $CDCl_3$.

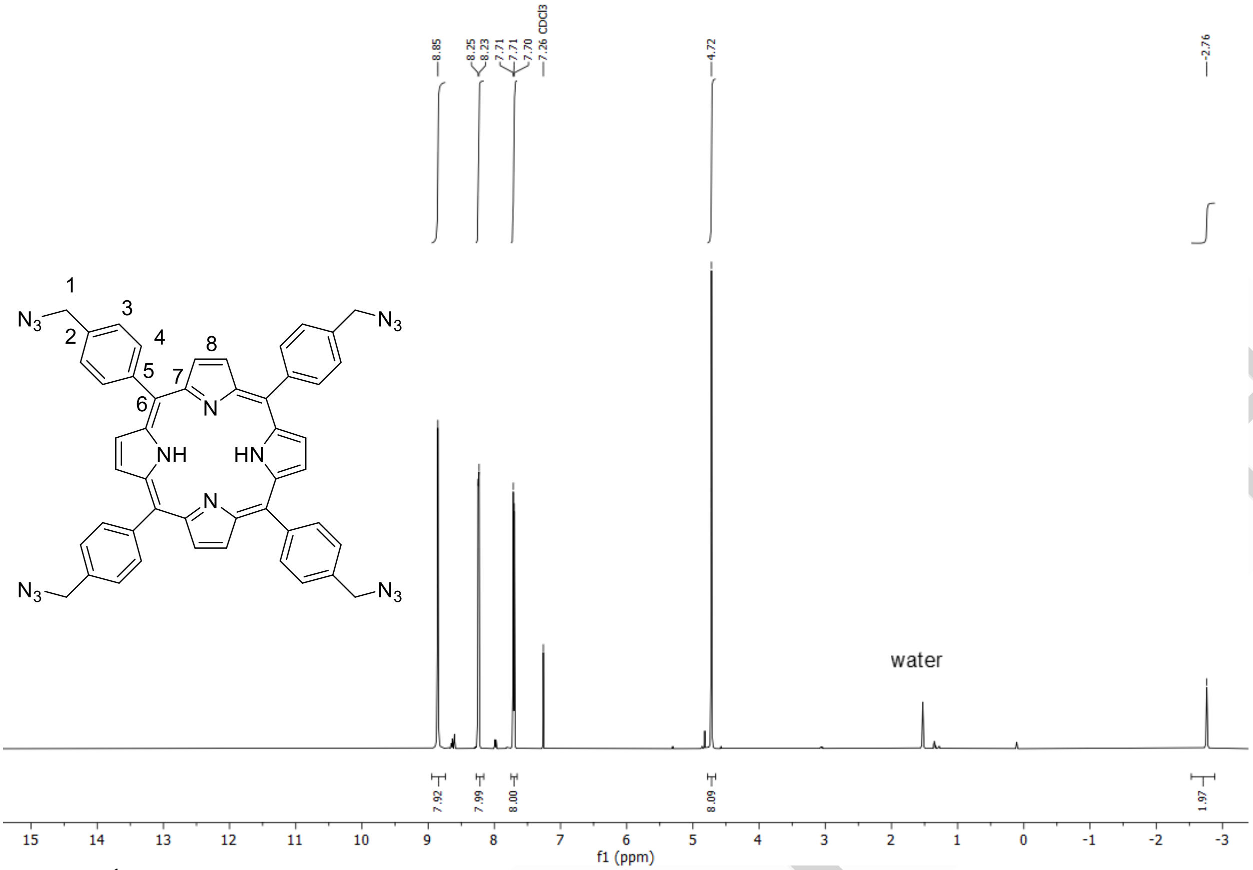

Figure S5. ^{1}H NMR spectrum of compound **5** in $CDCl_3$.

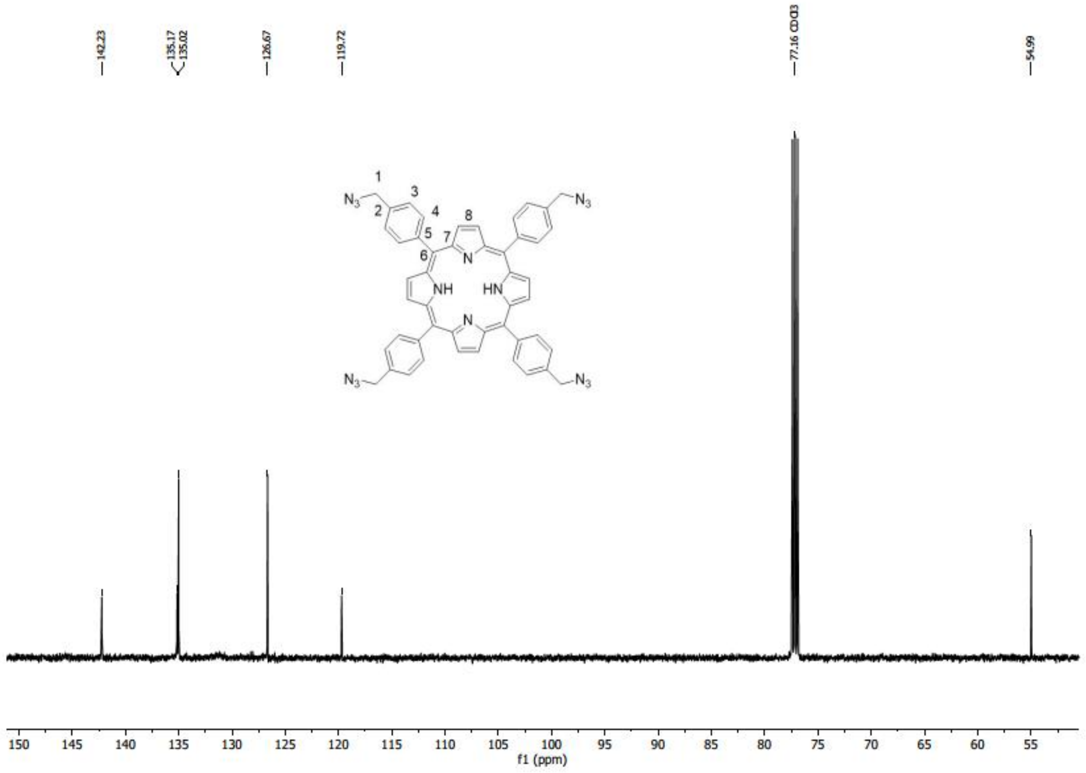

Figure S6. ^{13}C-NMR of compound **5** in $CDCl_3$.

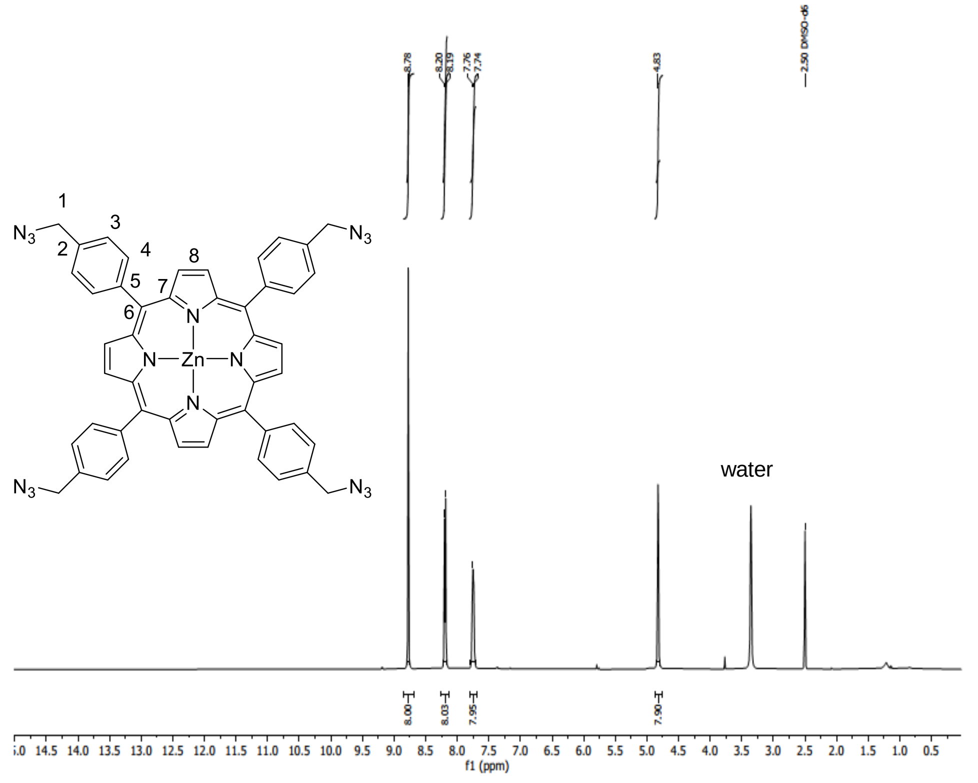

Figure S7. ^{1}H NMR spectrum of compound **Zn-5** in DMSO-d6**.**

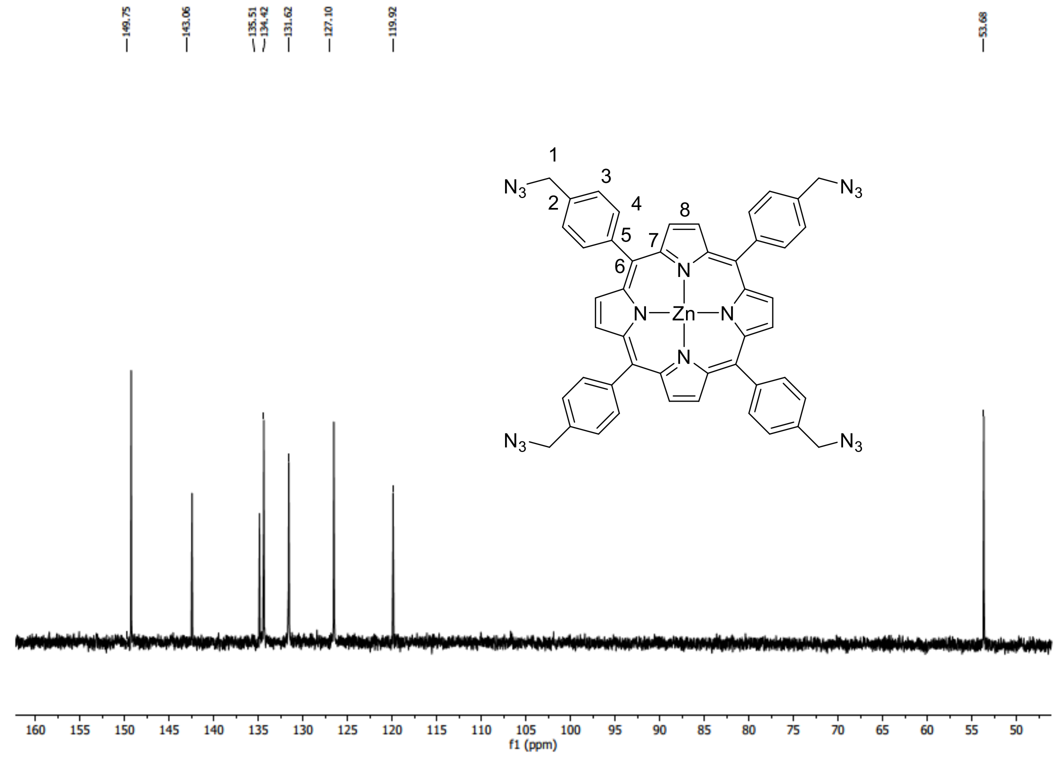

Figure S8. ^{13}C NMR spectrum of compound **Zn-5** in DMSO d6**.**

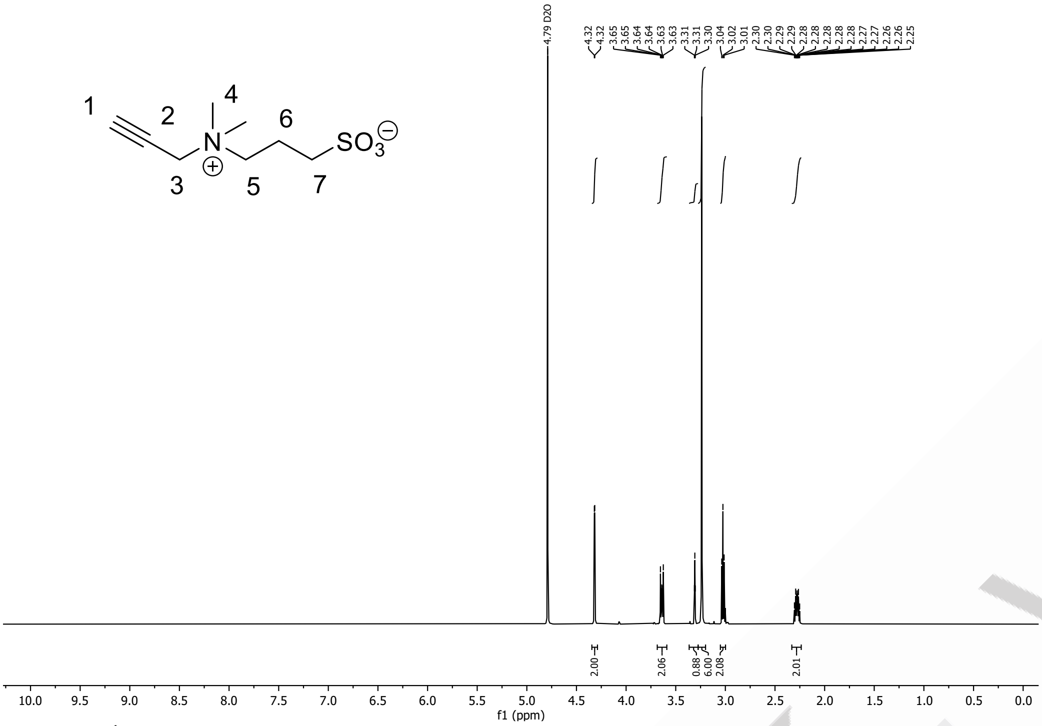

Figure S9. ^{1}H NMR spectrum of compound **6** in D_2O**.**

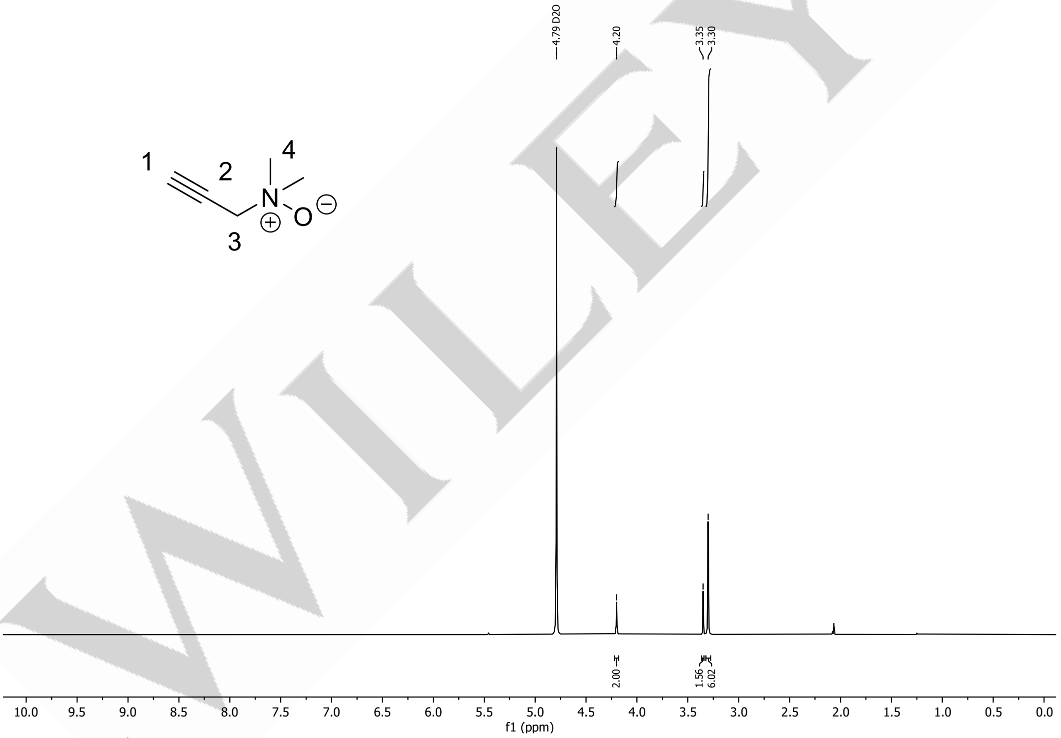

Figure S10. ^{1}H NMR compound of compound **7** in D_2O**.**

WILEY-VCH

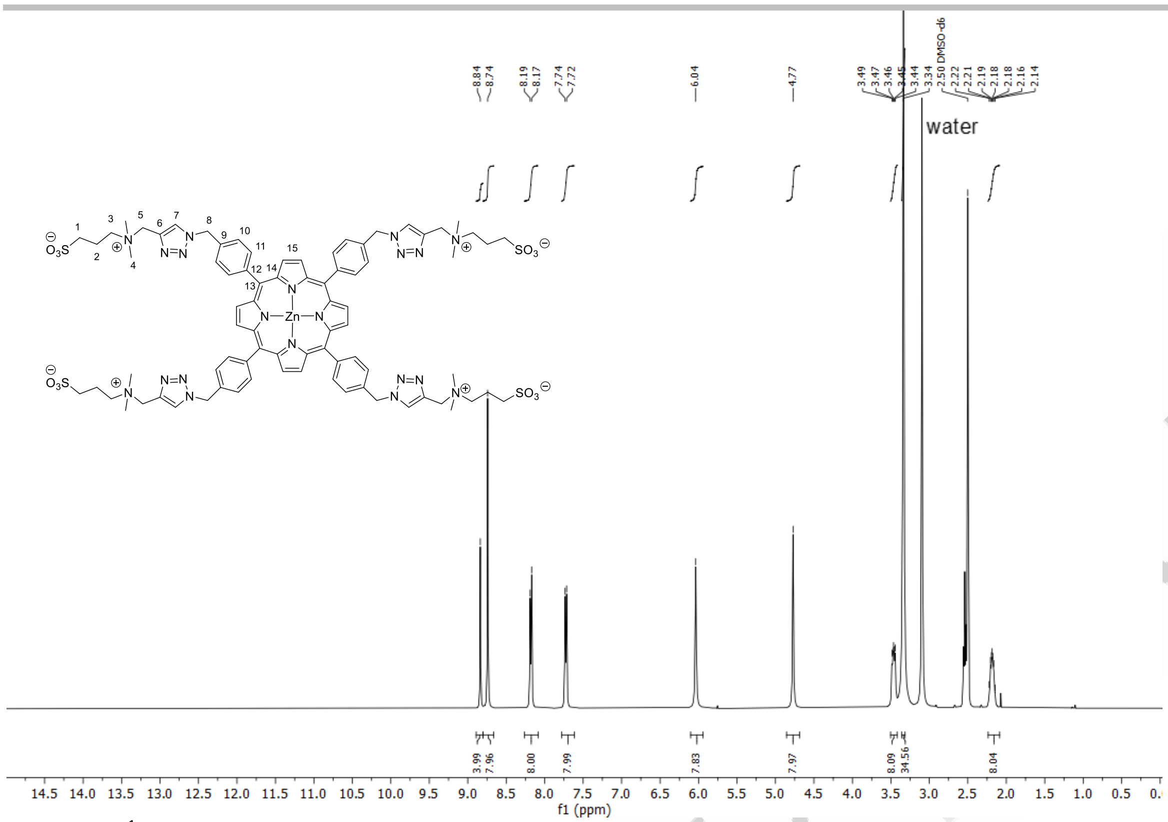

Figure S11. ^{1}H NMR spectrum of compound **Zn-8** in D_2O.

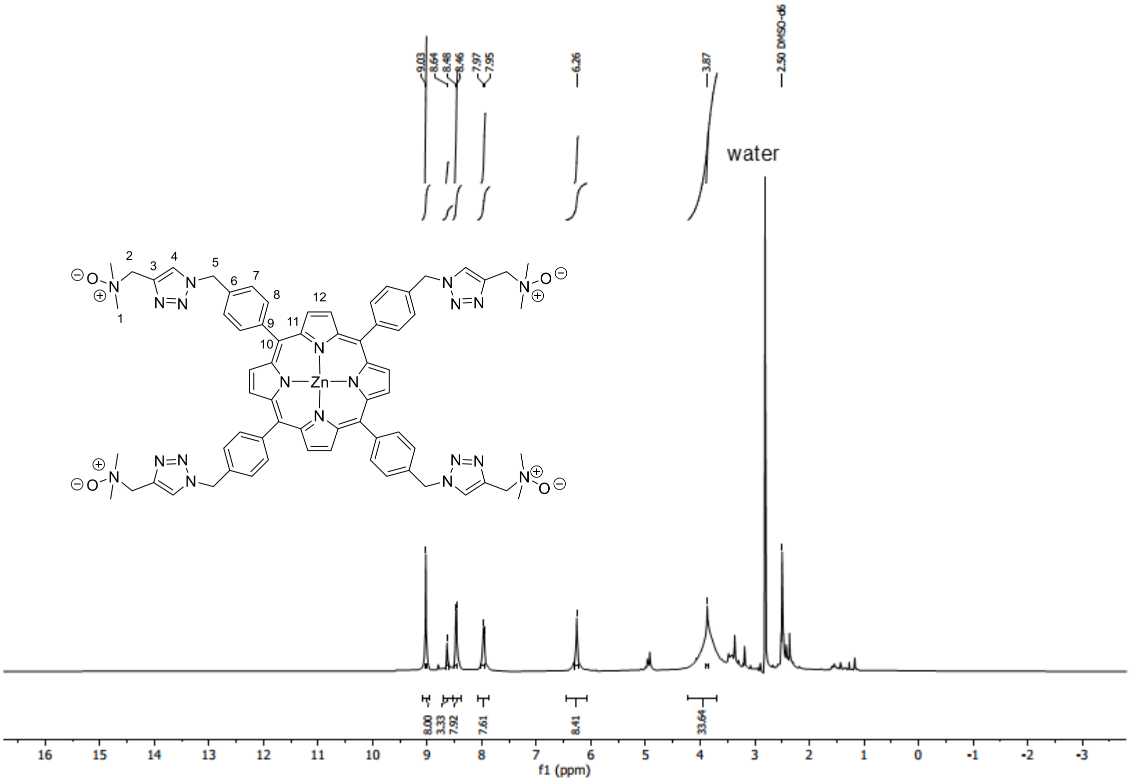

Figure S12. ^{1}H NMR spectrum of compound **Zn-9** in D_2O.

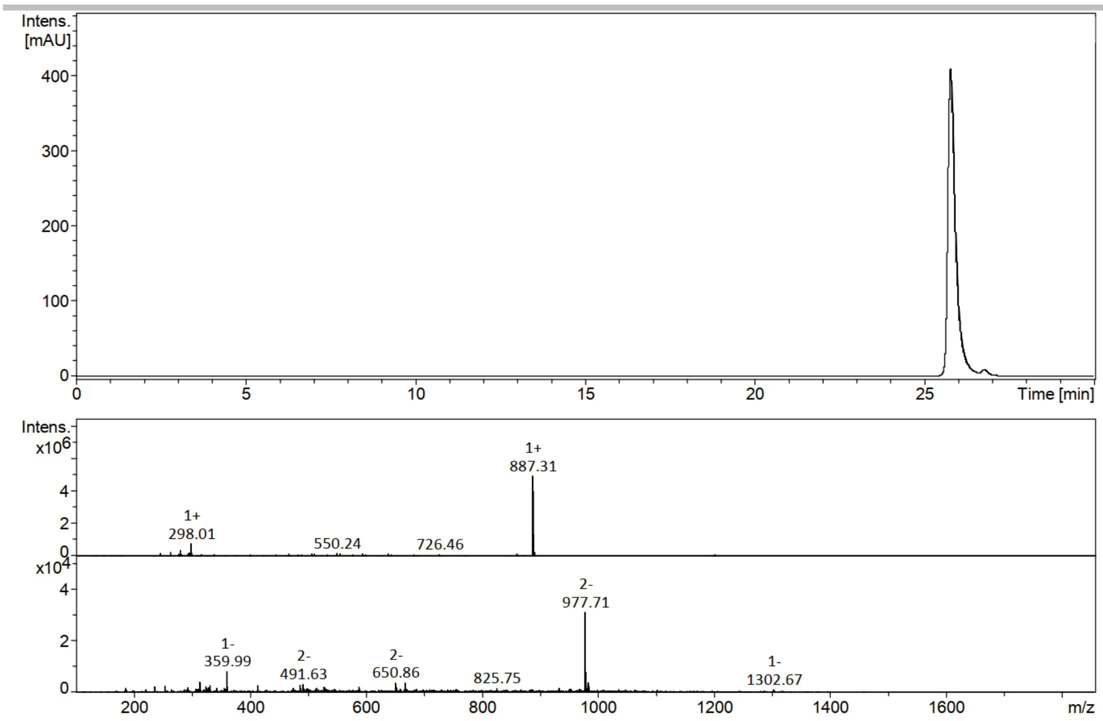

Figure S13. LC-MS chromatogram of compound **Mn-5**, top: UV-trace, detection wavelength 254 nm, middle: MS in positive mode, bottom: MS in negative mode**.**

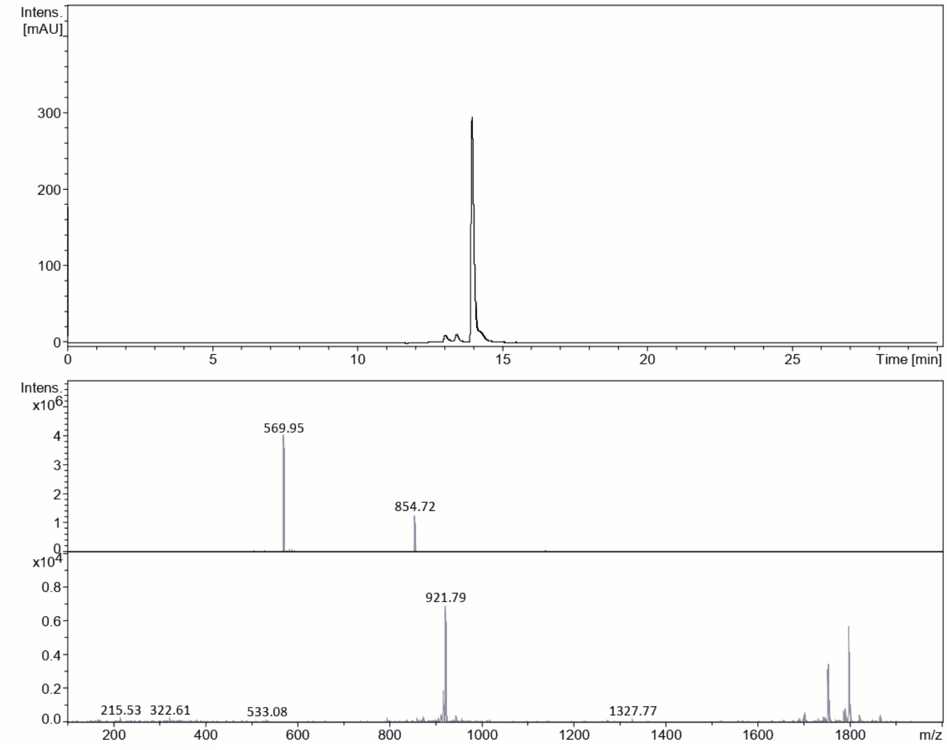

Figure S14. LC-MS chromatogram of compound **Mn-8**, top: UV-trace, detection wavelength 254 nm, middle: MS in positive mode, bottom: MS in negative mode**.**

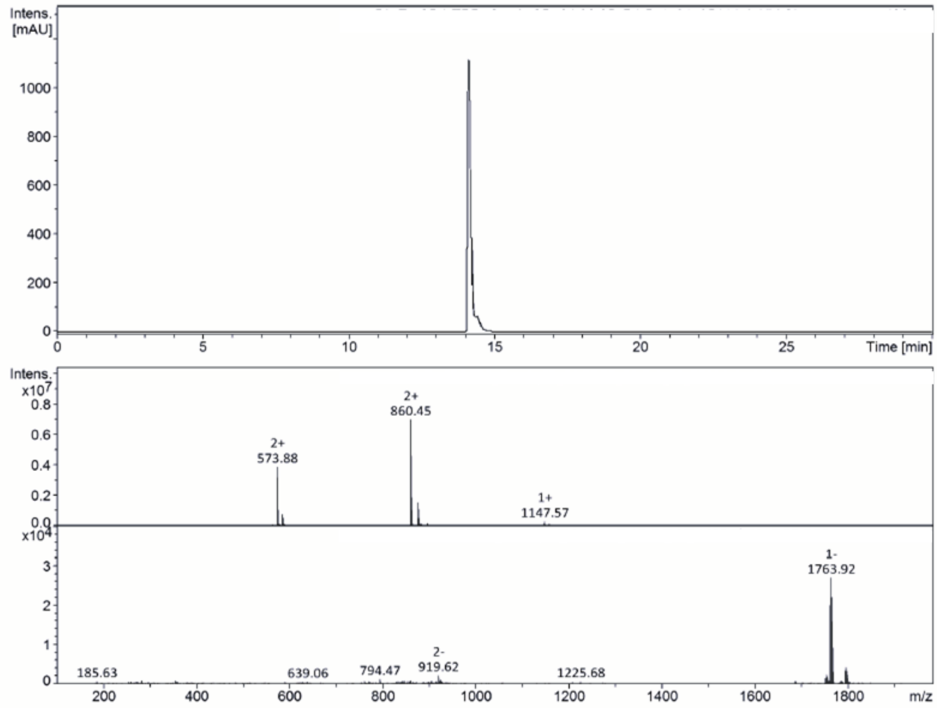

Figure S15. LC-MS chromatogram of compound **Zn-8**, top: UV-trace, detection wavelength 254 nm, middle: MS in positive mode, bottom: MS in negative mode**.**

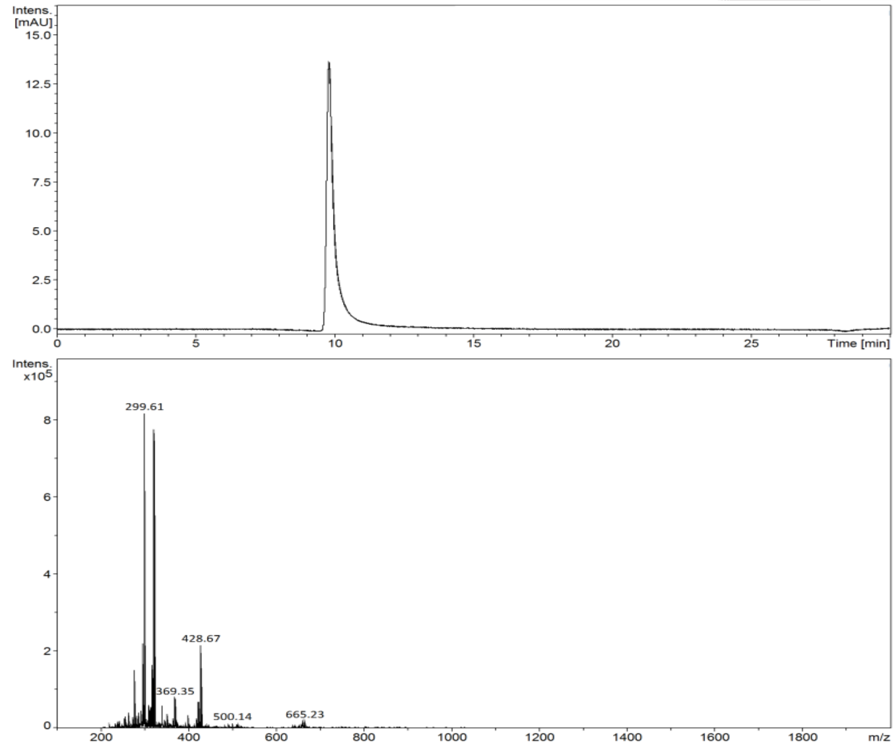

Figure S16. LC-MS chromatogram of compound **Mn-9**, top: UV-trace, detection wavelength 254 nm, bottom: MS in positive mode. Weak ionization was achieved in positive mode only with this compound.

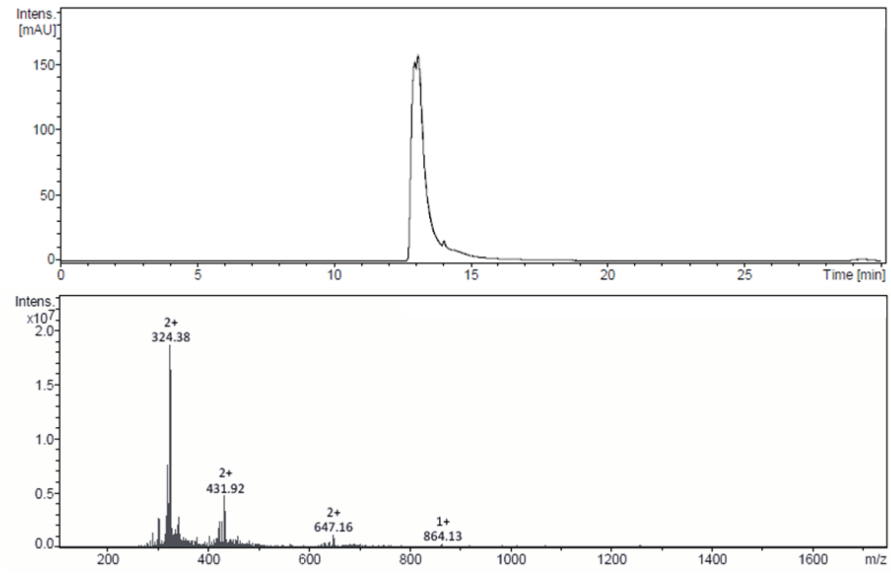

Figure S17. LC-MS chromatogram of compound **Zn-9**, top: UV-trace, detection wavelength 254 nm, bottom: MS in positive mode. Weak ionization was achieved in positive mode only with this compound.

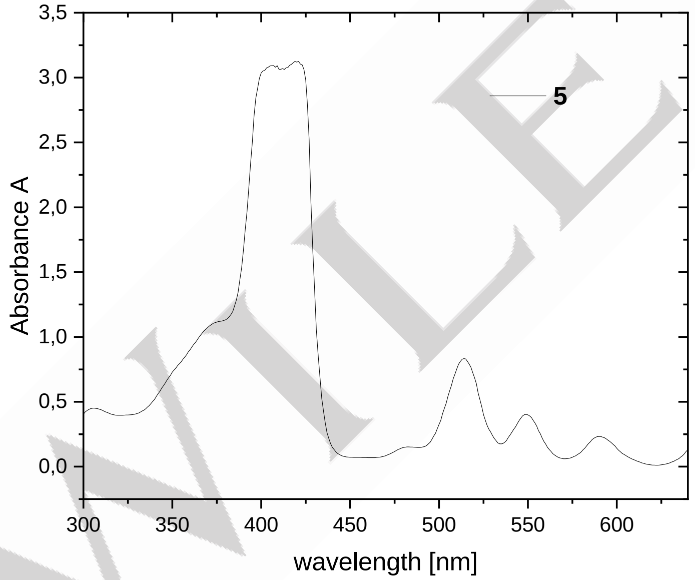

Figure S18. UV-vis Spectra of compound **5** in DMF.

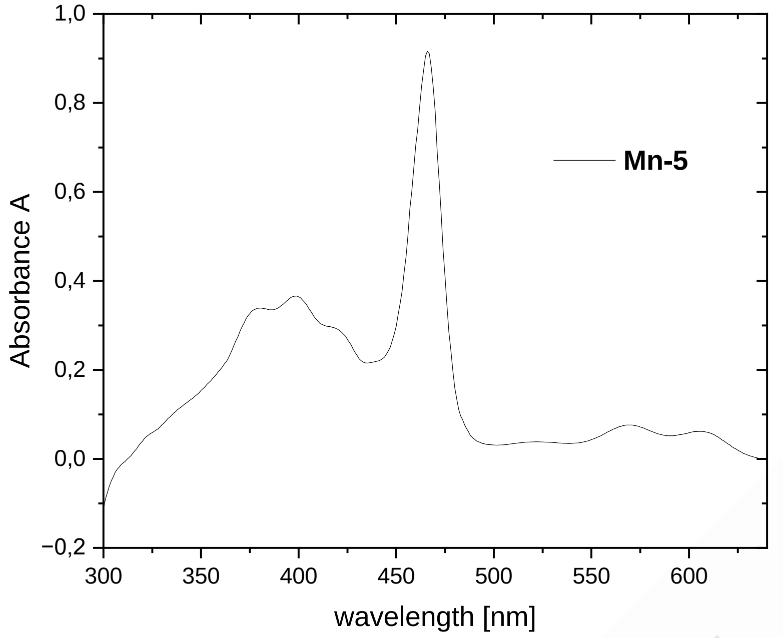

Figure S19. UV-VIS Spectra of compound **Mn-5** in DMF.

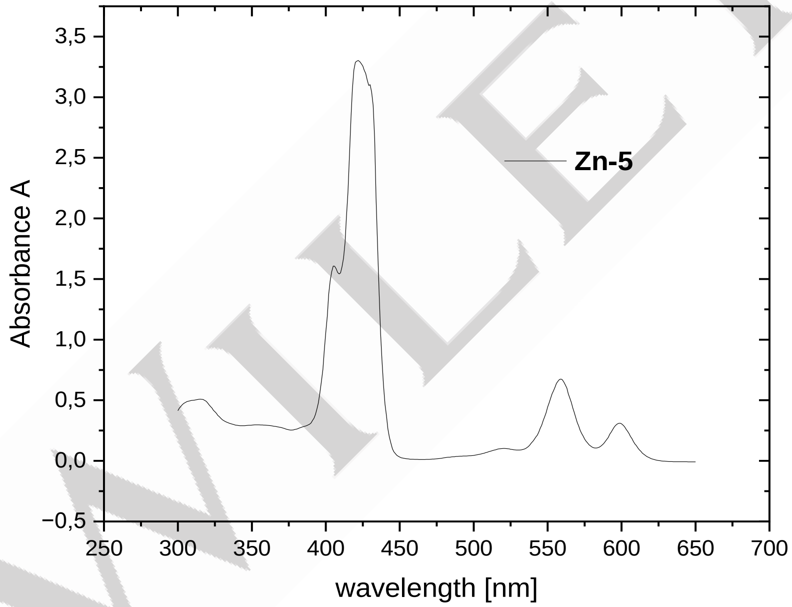

Figure S20. UV-VIS Spectra of compound **Zn-5** in DMF.

WILEY-VCH

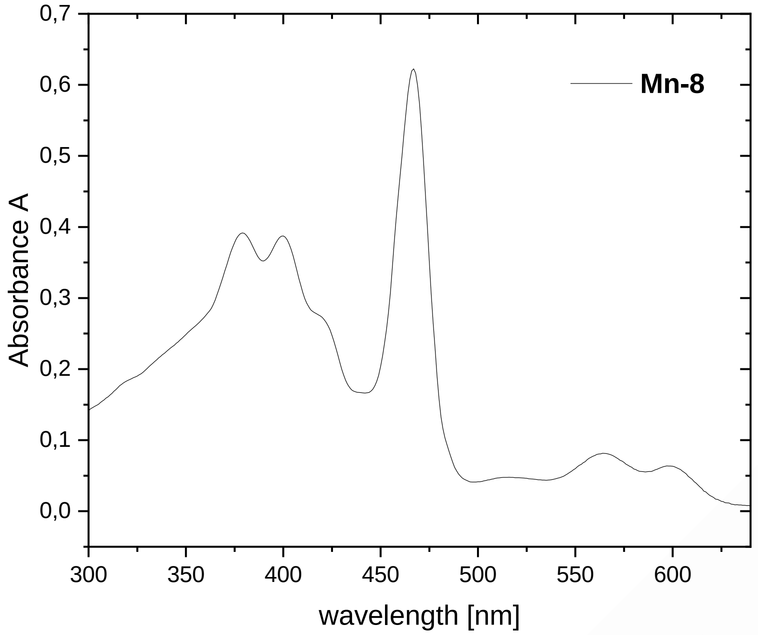

Figure S21. UV-VIS Spectra of compound **Mn-8** in H_2O.

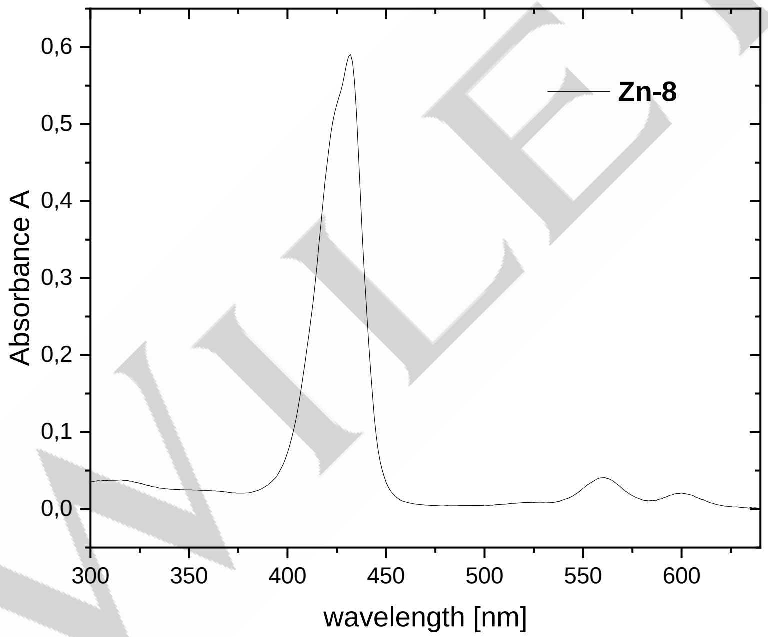

Figure S22. UV-VIS Spectra of compound **Zn-8** in H_2O.

WILEY-VCH

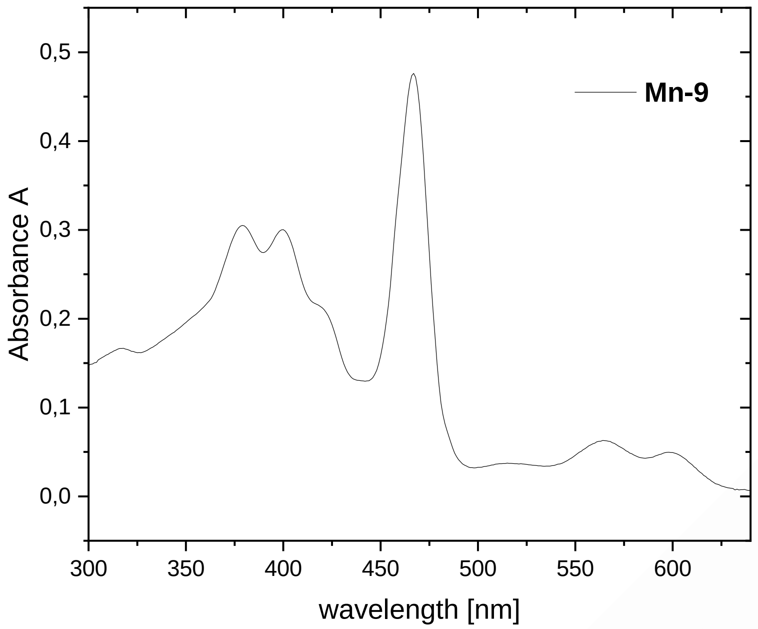

Figure S23. UV-VIS Spectra of compound **Mn-9** in H_2O.

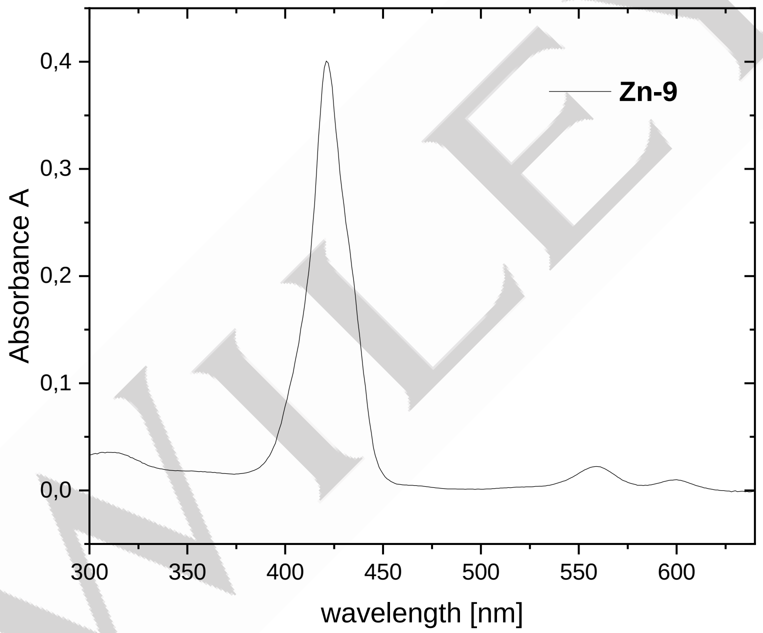

Figure S24. UV-VIS Spectra of compound **Zn-9** in H_2O.

WILEY-VCH

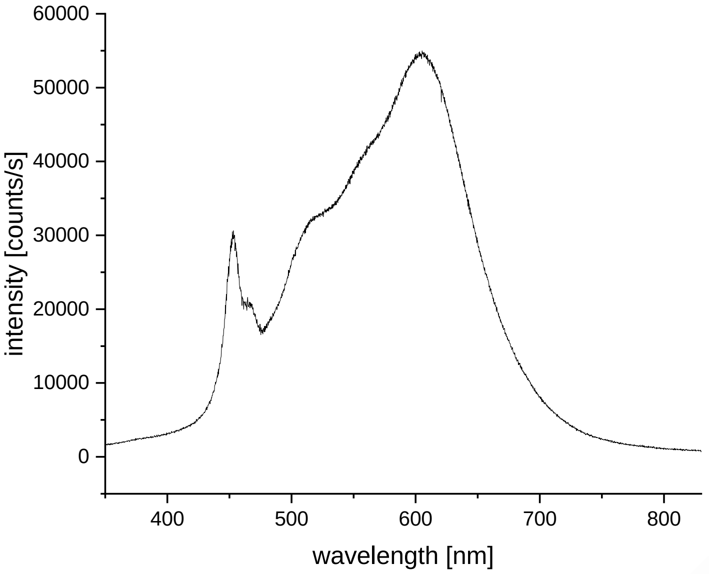

Figure S25. Spectral data of the LED utilized in singlet-oxygen assay.

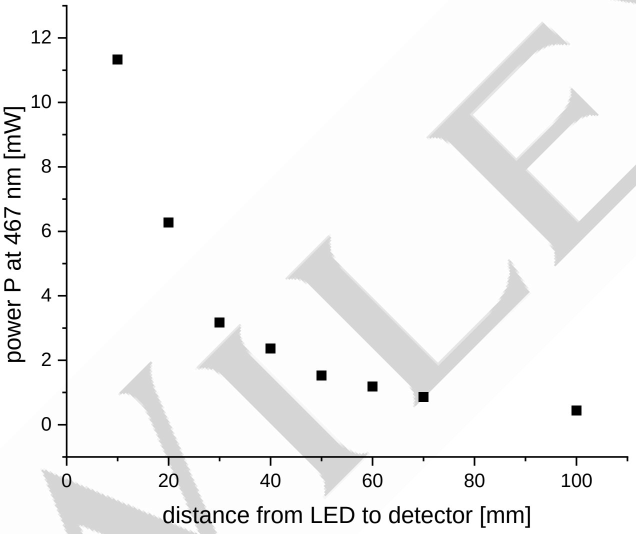

Figure S26. Detected power in mW at 467 nm at various distances from the LED.

WILEY-VCH

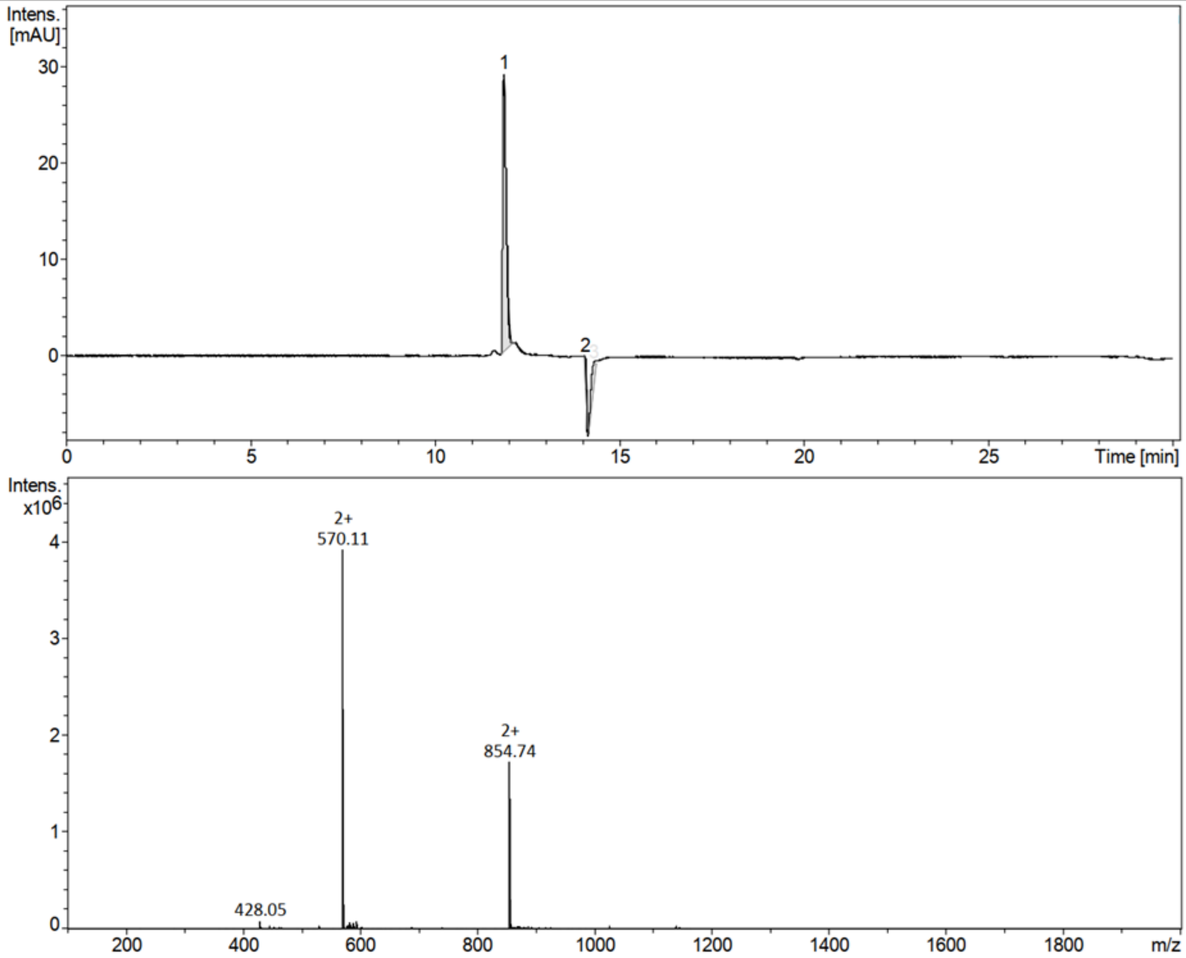

Figure S27: LC-MS chromatogram of porphyrin **Mn-8** following ultracentrifugation (30 kDa MWCO) from human serum, incubation time t_0. Integrated peak area 188.85 units. top: UV-trace, detection wavelength 254 nm, bottom: MS in positive mode**.**

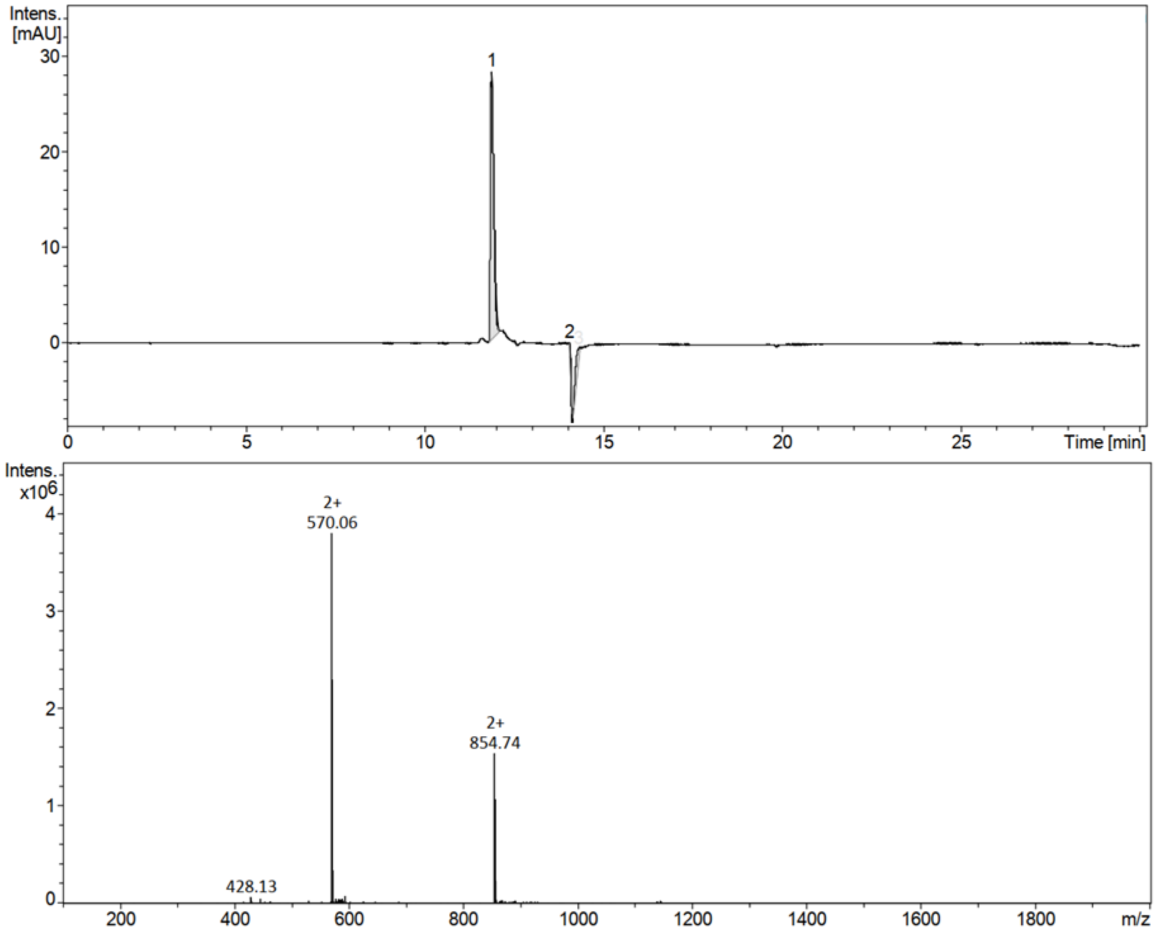

Figure S28: LC-MS chromatogram of porphyrin **Mn-8** following ultracentrifugation (30 kDa MWCO) from human serum, incubation time t_{21}. Integrated peak area 184.56 units. top: UV-trace, detection wavelength 254 nm, bottom: MS in positive mode**.**

WILEY-VCH

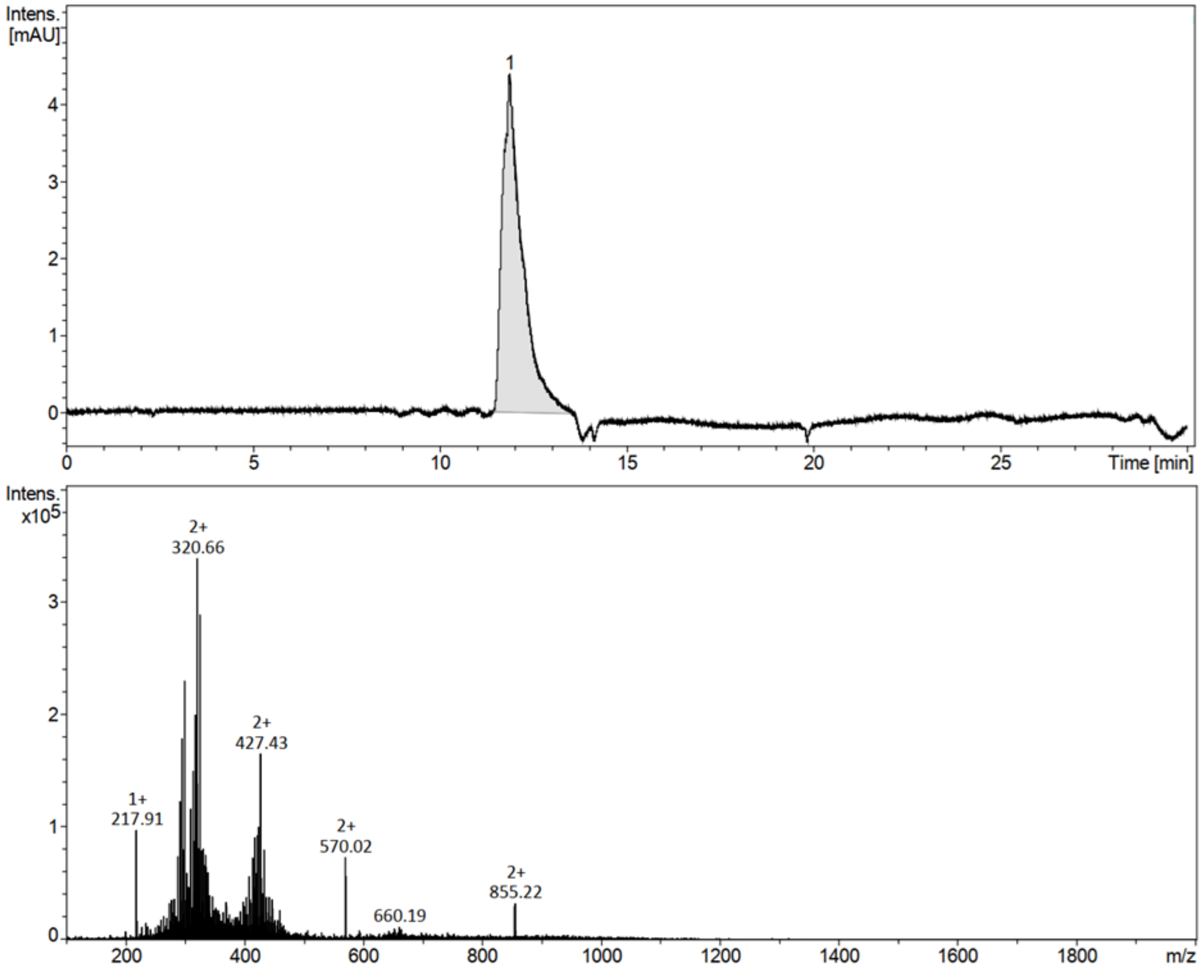

Figure S29: LC-MS chromatogram of porphyrin **Mn-9** following ultracentrifugation (30 kDa MWCO) from human serum, incubation time t_0. Integrated peak area 172.67 units. top: UV-trace, detection wavelength 254 nm, bottom: MS in positive mode**.**

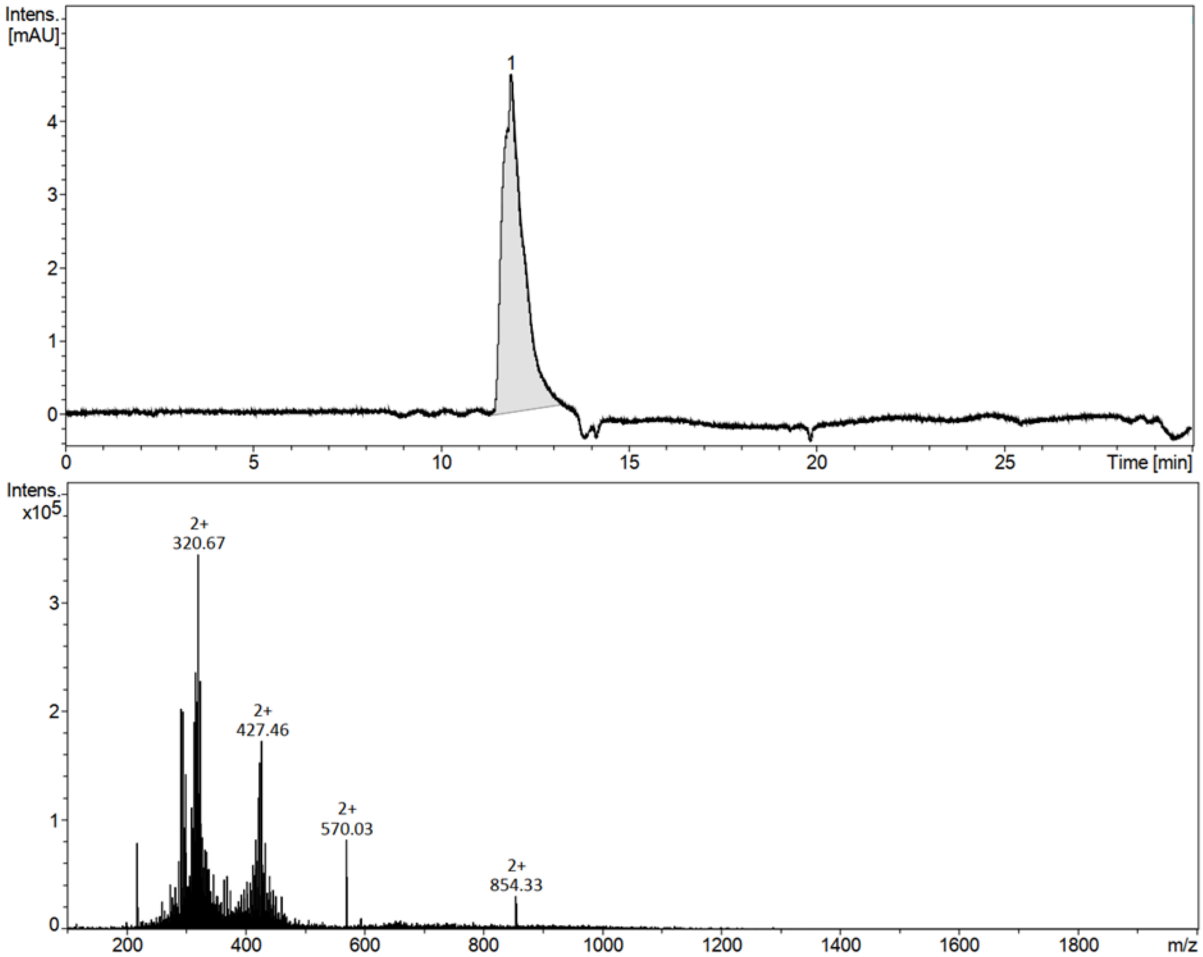

Figure S30: LC-MS chromatogram of porphyrin **Mn-9** following ultracentrifugation (30 kDa MWCO) from human serum, incubation time t_{21}. Integrated peak area 160.36 units. top: UV-trace, detection wavelength 254 nm, bottom: MS in positive mode**.**

References

[1] L. Le Pleux, Y. Pellegrin, E. Blart, F. Odobel, A. Harriman, Long-Lived, Charge-Shift States in Heterometallic, Porphyrin-Based Dendrimers Formed via Click Chemistry*, J. Phys. Chem. A.* **2011**, *115*, 5069.

[2] S. L. Niu, G. Ulrich, P. Retailleau, J. Harrowfield, R. Ziessel, New insights into the solubilization of Bodipy dyes*, Tetrahedron Lett* **2009**, *50*, 3840.

[3] A. Galan, G. Gil-Ramirez, P. Ballester, Kinetic stabilization of N,N-dimethyl-2-propyn-1-amine N-oxide by encapsulation*, Org. Lett.* **2013**, *15*, 4976.
